# Characterization of bacterial communities associated with the exotic and heavy metal tolerant wetland plant *Spartina alterniflora*

**DOI:** 10.1038/s41598-020-75041-5

**Published:** 2020-10-22

**Authors:** Ying Yang, Jian Ding, Yulang Chi, Jianjun Yuan

**Affiliations:** 1 College of Oceanology and Food Science, Quanzhou Normal University, Quanzhou, 362000 China; 2 State Key Laboratory of Marine Environmental Science, College of Ocean and Earth Sciences, Xiamen University, Xiamen, 361005 China; 3 Sunshine Guojian Pharmaceutical (Shanghai) Co., Ltd, Shanghai, China

**Keywords:** Ecology, Environmental sciences

## Abstract

Heavy metal pollution has seriously disrupted eco-balance and transformed estuaries into sewage depots. Quanzhou bay is a typical heavy metal-contaminated estuary, in which *Spartina alterniflora* has widely invaded. Plant-associated microbial communities are crucial for biogeochemical cycles, studies of which would be helpful to demonstrate the invasion mechanisms of plants. Meanwhile, they are indispensable to phytoremediation by enhancing the heavy metal tolerance of plants, facilitating heavy metal absorption rate and promoting growth of plants. In the present study, *S. alterniflora*-associated rhizo- and endobacterial communities from 3 experimental sites were investigated by 454-pyrosequencing. Heavy metal screening generated 16 culturable isolates, further biochemical assays suggested these clones possess various abilities such as phosphate solubilization, indole-3-acetic acid (IAA) production and 1-aminocyclopropane-1-carboxylate (ACC) deaminase production to accelerate heavy metal uptake and growth of the host. This study revealed the bacterial community structures and characterized the predominant resident bacterial strains of *S. alterniflora-*associated rhizo- and endobacteria under heavy metal stress, and isolated several bacterial species with potential ecological function.

## Introduction

According to previous studies, plant invasion was believed to be not only a threat to native biodiversity, but also could affect ecosystem functioning and process through a variety of mechanisms, such as reducing plant and animal biodiversity, altering wetland hydrology and changing carbon or nitrogen cycling^1^. At present, two hypotheses of plant invasion mechanisms are commonly accepted: biological interactions, including resource availability and enemy release^2^. The invasion of exotic plant might change the original microbial community structure in the sediments. For example, *Spartina alterniflora*, native to the east coast of North America, is proved to affect the community structures of methanogens and sulfate-reducing bacteria in *Phragmites australis* vegetated sediments^3^. Meanwhile, plants (e.g., *Lepidium sativum*, *Oryza sativa* cultivars and maize) might also benefit from their associated rhizo- and endobacteria by enhancing their growth, productivity, stress resistance and even phytoremediation^4–6^.

Since the industrial revolution, heavy metal pollution has become a prevalent environmental threat^7^. Urban estuaries have been regarded as suitable places for the disposal of industrial and domestic wastes due to their proximity to the open ocean^7,8^. After long period of accumulation, natural biotic and physio-chemical balance of previously pristine system is disrupted and transformed into sewage depots. Unlike organic pollutants, heavy metal pollutants could not be removed by natural processes and start to move up the food chain once they accumulated in sediments. Moreover, the accumulation of potentially toxic heavy metals might also have a detrimental influence on physical metabolism, activity, biomass, diversity and composition of plant and microbial communities^9^. In order to survive under heavy metal stress, plants develop a series of coping strategies such as reducing water content, producing antioxidases and altering osmotic adjustment substances, to relieve the damage of heavy metal stress^10^. Wetland plants such as *P. australis*, *Typha capensis* and *Spartina maritima* can accumulate heavy metal from environment, and are considered as potential phytoremediators and indicators of heavy metal contamination^11^. Plant-associated microbes like endophytic and rhizosphere bacteria can enhance plant growth and increase the absorption of heavy metal at the same time. The plant-associated bacteria migrate from the bulk soil to the rhizosphere of living plant and aggressively colonize the rhizosphere and roots of plants^12^. Only a few of plants have been well studied regarding their rhizosphere and endophytic biology. Among the rhizo- and endobacteria involved in interactions between plants and metal-contaminated soil, the plant growth promoting bacteria (PGPB) are important. The metabolites released by PGPB (e.g., siderophores, biosurfactants, organic acids, plant growth regulator, etc.) can alter the uptake of heavy metals indirectly and directly^13^.

*Spartina alterniflora* was introduced to China in 1979 and had rapidly invaded the salt marshes on the east coast of China^14^. Its displacement of the native species had caused a number of ecological impacts^14,15^. Several studies investigated the effects of *S. alterniflora* on ecosystem^15–17^ and its rhizo- and endobacterial diversity^18^, etc. However, little was known about the rhizo- and endobacterial community of *S. alterniflora* under heavy metal stress.

The Quanzhou bay is a typical estuary where the water quality and ecological health of the system have been markedly polluted by inputs from the upstream regions. Industrial and domestic wastewater alongside the Luoyang River and Jinjiang River was discharged into Quanzhou bay, forming the “black and white” appearance of water body^19^. In Quanzhou bay estuary, the average salinity of soil is partially different in different sections, the average pH in soil has a small variation range (from 6.92 to 7.66) and the total organic carbon level vary from 0.81 to 1.88%^20,21^. Surveys on sediment and water quality indicated that heavy metals in the wastewater were the principal pollutant in Quanzhou bay^20^. Despite severe heavy metal pollution, *S. alterniflora* had widely invaded Quanzhou bay, offering an opportunity to understand the influence of rhizo- and endobacteria on the niche advantages of *S. alterniflora*.

Our study investigated the *S. alterniflora*-associated rhizo- and endobacterial community structures and diversity from 3 sampling sites, one (site 3) of which is allocated at an oil terminal. To analysis *S. alterniflora*-associated bacterial community composition and diversity, both culture-independent and traditional culture-dependent methods were applied. Biochemical assays were performed to screen for the heavy metal-resistant bacteria. These results would provide the basis for further study on the structure and function of rhizosphere and endophytic bacterial communities associated with *S. alterniflora* under heavy metal pollution, and established rhizo- and endobacterial libraries as references for future microbial application.

## Results

### Differences of microbial diversity between rhizosphere soil and roots of *S. alterniflora*

**Figure 1:**
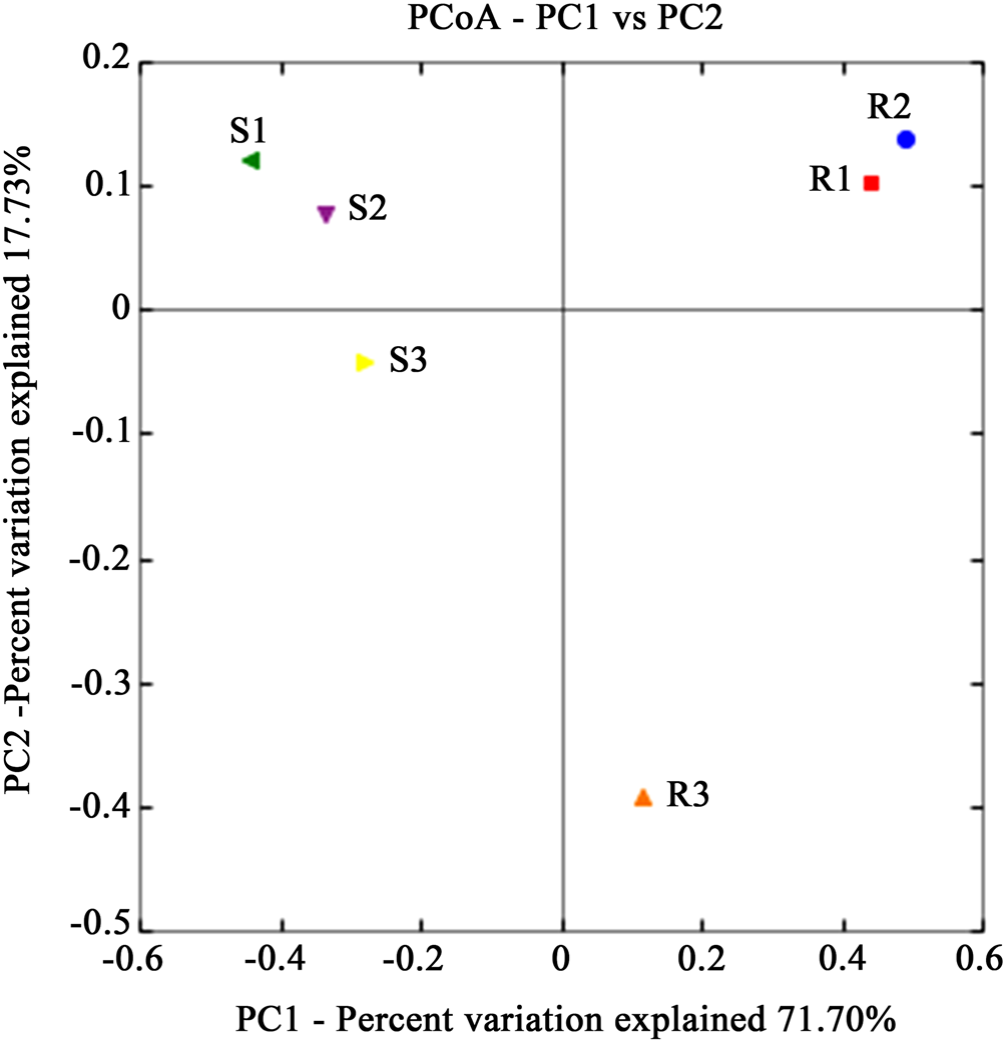
Principal component analysis of all 454 libraries with weighted UniFrac. X-axis, 1st principal component and Y-axis, 2nd principal component. Numbers in brackets represents contributions of principal components to differences among samples. A dot represents each sample. Figure was drawn using R (v3.1.1, https://www.datavis.ca/R/).

The heavy metal content of sediments and rhizosphere soil was assessed. The results showed that there are different degrees of heavy metal pollution in the 3 sampling sites (see Supplementary Table S1 online). To investigate the rhizo- and endobacterial diversity associated with *S. alterniflora* under different degrees of heavy metal contamination, we applied 454-pyrosequencing targeting V4 region of 16S rRNA. The 454-pyrosequencing generated over 155,346 reads. In total, 143,009 reads were obtained after quality control (average 29,403 reads per rhizosphere sample and 16,309 reads per endophytic sample). Trimmed sequences generated 14,537 operational taxonomic units (OTUs). On average, 4629 and 1191 OTUs per rhizosphere and endophytic sample were detected, respectively. Based on the rarefaction data (see Supplementary Fig. S1 online) and diversity indices of 454 tag sequences (Table 1), the sequencing depth could not completely cover full diversity of rhizosphere soil, indicating that the microbial community of rhizosphere soil was more diverse than that of the plant roots. It was worth noting that S3 hosted the most diverse and least abundant rhizobacterial community, while R3 displayed a reversed pattern. Weighted PCoA plot (Fig. 1) showed that R1/R2 and S1/S2 form clusters respectively, while S3/R3 was distant. The results suggested that both rhizo- and endobacterial communities were specific in geographical location.

**Table 1:** Database evaluation and diversity estimates of 454-pyrosequencing libraries.

Sample ID	Total of OTUs	Chimeras	Archaea of OTUs	No_archaea	Evenness (E)	Pielou index (J_gi_)	Shannon index (H′)	Simpson index (D)	Margalef index (d_Ma_)	Menhinick index (R_2_)	Coverage (C)	PIE	Chao 1
S1	37,415	184	2	37,229	0.776	0.991	8.434	0.991	652.923	35.027	0.902	0.991	10,066.347
S2	23,629	74	1	23,554	0.844	0.996	9.182	0.996	525.556	33.663	0.891	0.996	7643.259
S3	27,472	49	0	27,423	0.858	0.997	9.706	0.997	547.053	33.014	0.909	0.997	7697.706
R1	25,130	12	0	25,118	0.812	0.995	7.685	0.995	117.486	11.280	0.977	0.995	2232.554
R2	9242	14	0	9228	0.811	0.990	6.990	0.989	105.610	9.872	0.964	0.989	1012.130
R3	14,617	34	0	14,583	0.738	0.974	7.025	0.973	179.699	14.022	0.947	0.973	2364.175

### Composition of the rhizo- and endobacterial communities

**Figure 2:**
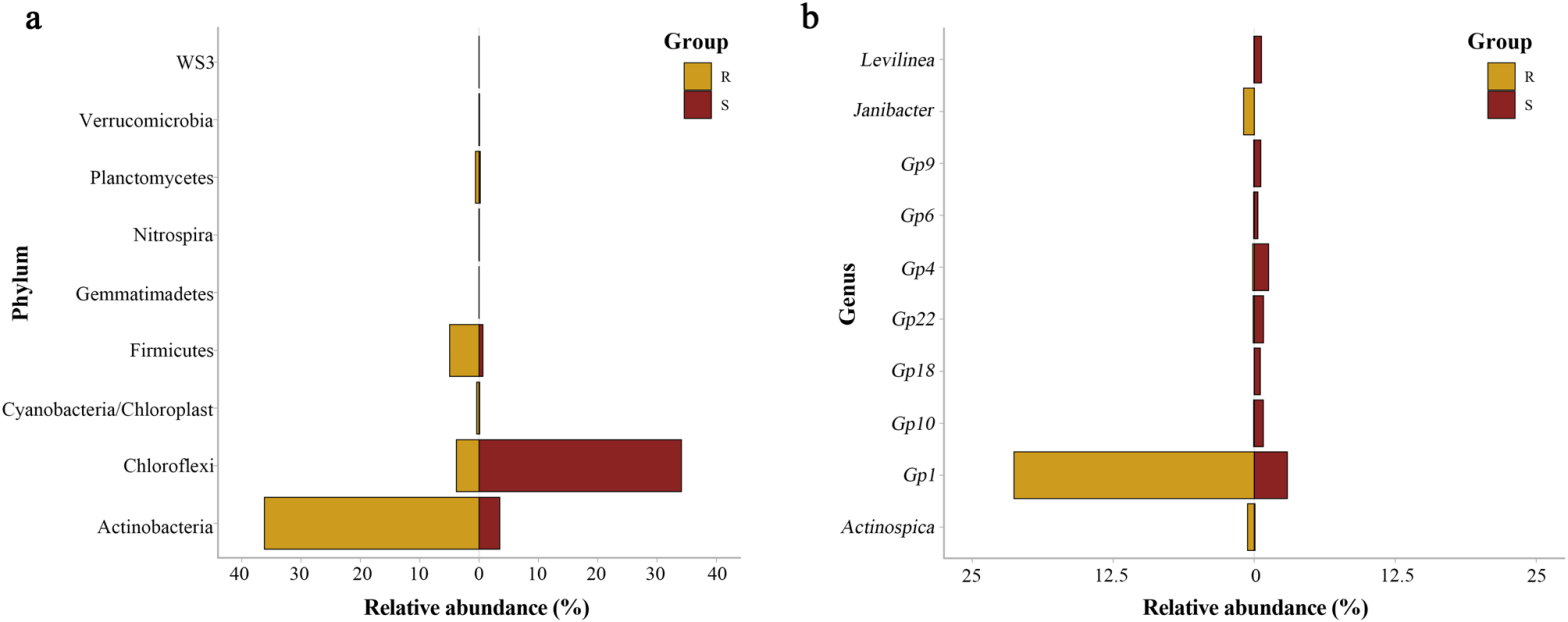
Phyla (**a**) and genera (**b**) with significant differences in relative abundance in rhizosphere soil and roots. *R* roots of *Spartina alterniflora*, *S* rhizosphere soil of *S. alterniflora*. Figure was drawn using R (v.3.1.1, https://www.datavis.ca/R/).

The composition of rhizo- and endobacterial communities was highly distinct (see Supplementary Fig. S2 online), and the phyla and genera (partial) with significant differences in relative abundance in rhizosphere soil and roots were shown in Fig. 2. The relative abundance of Actinobacteria and Firmicutes in root was significantly higher than that in rhizosphere soil. Chloroflexi constituted the majority of sequences in rhizosphere soil but showed less abundance in endophytic root. Acidobacteria was the second abundant phylum in both samples. The third abundant phylum allocated to Proteobacteria (12.69 ± 0.58% in soil, 9.66 ± 6.13% in root). Surprisingly, Actinobacteria seemed to concentrate in root (39.27 ± 8.74%) but not in rhizosphere soil (4.18 ± 2.80%). At genus level (see Supplementary Fig. S3 online), an uncultured group from Anaerolineaceae was dominant in rhizosphere soil (44.68 ± 15.11%), followed by *Nitrospina* (1.86 ± 0.79%), *Acidothermus* (1.78 ± 1.74%). For R1 and R2, *Acidothermus* appeared to be the dominant group (19.62 ± 12.01%) followed by *Acidobacterium* (13.53 ± 7.93%). Interestingly, R3 had a distinguished pattern, which showed the same dominant genus as rhizosphere soil (20.33%) followed by uncultured Propionibacteriaceae (18.50%) and *Desulfovibrio* (8.38%).

### Differences of rhizo- and endobacterial community structure

**Figure 3:**
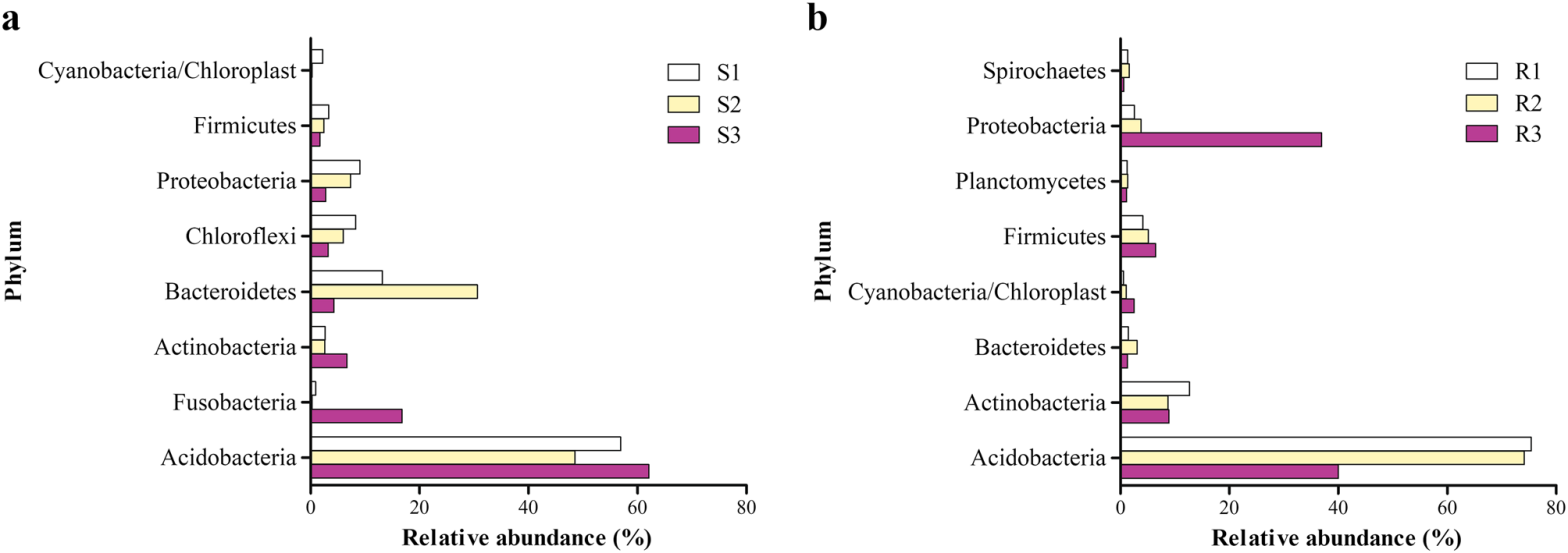
Dominant phyla (relative abundance > 1%) in rhizobacterial community (**a**) and root endobacterial community (**b**). *R* roots of *Spartina alterniflora*, *S* rhizosphere soil of *S. alterniflora*. Figure was drawn using GraphPad (v5.01).

**Figure 4:**
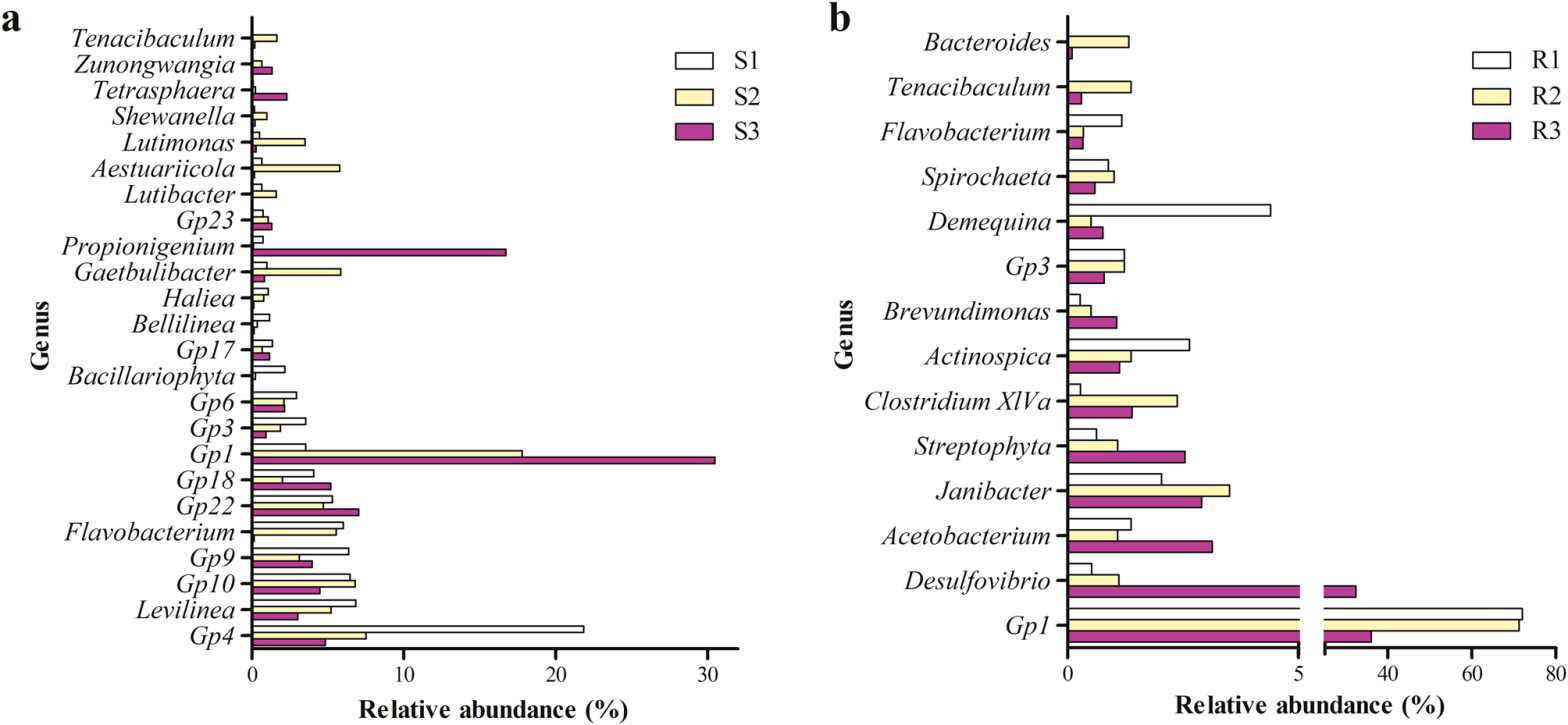
Dominant genera (relative abundance > 1%) in rhizobacterial community (**a**) and root endobacterial community (**b**). *R* roots of *Spartina alterniflora*, *S* rhizosphere soil of *S. alterniflora*. Figure was drawn using GraphPad (v5.01).

For a better understanding of rhizo- and endobacterial communities, we screened out 8 phyla and 24 genera that had relative abundance > 1% in at least one site (Figs. 3, 4). Overall, rhizobacterial communities (S1, S2 and S3) were dominated by Chloroflexi. Acidobacteria, Actinobacteria and Fusobacteria were found enriched in S3 compared to S1 and S2. Actinobacteria was predominant in all rhizosphere samples. *Propionigenium,* and Acidobacteria_*Gp1* were significantly enriched in S3. Notably, while *Propionigenium* occupied a large proportion of S3, it was barely found in S1 and S2. With the same screening strategy, 8 phyla and 14 genera were obtained in endobacterial database (Figs. 3, 4). Actinobacteria predominated R1 and R2, while Proteobacteria and Acidobacteria together made up majority of R3. Relative proportion of Proteobacteria, Firmicutes and Cyanobacteria/Chloroplast were higher in R3. Further analysis showed the R1 and R2 share similar bacterial community structure with Acidobacteria_*Gp1* as the dominant genus. R3 was mainly consisted of members from Acidobacteria_*Gp1*, *Desulfovibrio, Janibacter*, *Acetobacterium* and *Streptophyta*.

### 16S rRNA sequencing

By using the same environmental DNA extracts and clones obtained from traditional cultivation method, small-scale verification conducted in lab by full-length 16S rRNA sequencing revealed a slightly different pattern. Taxonomic characterization suggested that most of the isolates were Firmicutes (in soil and endophytic leaves) and Proteobacteria (in endophytic roots). Results turned out to be the subsets of 454-pyrosequencing dataset, indicating 454-pyrosequencing an efficient way to get better insight of microbial community structure since large proportion of bacteria could be underrepresented due to disadvantages of traditional methods. But several genera like *Xanthobacter, Methanoplanus, Exiguobacterium, Rheinheimera, Sinorhizobium, Yokenella, Planococcus,* etc. that were believed to possess multiple potential ecological functions were identified by full-length sequencing but showed no representation in 454-pyrosequencing (see Supplementary Tables S2 and S3 online). Sequences with low abundance might be underrepresented by high-throughput methods. Therefore, combination of traditional and next-generation sequencing strategies would provide us a comprehensive understanding of microbiology community under natural status.

### Identification and biochemical characterization of heavy metal resistant clones

Refer to previous studies on heavy metal content of wetland plants and preliminary experiments, proper concentrations were added for screening of heavy metal resistant endophytes (50 ppm Cu^2+^, 100 ppm Pb^2+^, 50 ppm Zn^2+^, 5 ppm Cd^2+^, 5 ppm Cr^6+^ and 30 ppm Ni^2+^). Most of the screened endophytes showed multiple resistances to different heavy metal, 5 isolates of them were able to grow on non-nitrogen medium. Biochemical characterization resulted in thirteen IAA producers, five ACC deaminase producer, one siderophore producer and one isolate that could solubilize phosphate (Table. 2). Blastn analysis of these sequences revealed that 34 of them were very similar to *Psychrobacter* sp. PRwf-1 (NR_074709), this isolate could tolerate 3 out of 5 examined heavy metal and also produce IAA, ACC deaminase. The second highest hits could be affiliated to *Lysinibacillus fusiformis* strain DSM 2898.

**Table 2:** Biochemical characterization of heavy metal resistant endobacteria.

Closest relatives	Total hits	Cu^2+^	Pb^2+^	Zn^2+^	Cd^2+^	Cr^6+^	Ni^2+^	Nitrogen fixation	IAA (10^−6^ mol/L)	Siderophore production	Siderophore units	As/Ar	ACC deaminase activity (2-ketobutyric acid umol/mg·h)	Phosphate solubilization
*Psychrobacter* sp. PRwf-1 (NR_074709)	34	✓	✓			✓			14.51 ± 0.57	–	–	–	0.25 ± 0.28	–
*Lysinibacillus fusiformis* strain DSM 2898 (NR_042072)	24	✓	✓			✓			7.12 ± 0.33	–	–	–	–	–
*Chryseobacterium daecheongense* strain CPW406 (NR_028980)	20			✓	✓		✓	✓	25.84 ± 0.39	–	–	–	–	–
*Lysinibacillus sphaericus* strain DSM 28 (NR_042073)	18	✓	✓			✓		✓	276.67 ± 0.45	–	–	–	0.01 ± 0.43	–
*Microbacterium oxydans* strain DSM 20578 (NR_044931)	18				✓		✓		15.39 ± 0.72	–	–	–	–	–
*Chryseobacterium gambrini* strain 5-1St1a (NR_042505)	17			✓	✓		✓		23.82 ± 0.89	–	–	–	–	–
*Paenibacillus xylanexedens* strain B22a (NR_044524)	9	✓						✓	17.31 ± 0.46	–	–	–	–	+
*Bacillus anthracis* strain Ames (NR_074453)	8				✓	✓	✓		21.79 ± 0.70	–	–	–	–	–
*Lactococcus lactis* subsp. lactis Il1403 strain IL1403 (NR_103918)	5					✓			–	–	–	–	–	–
*Bacillus megaterium* QM B1551 strain QM B1551 (NR_074290)	4				✓		✓		15.73 ± 0.48	+	4.22	1	1.53 ± 0.49	–
*Bacillus megaterium* strain IAM 13418 (NR_043401)	4				✓		✓		18.00 ± 0.34	–	–	–	0.76 ± 0.51	–
*Bacillus weihenstephanensis* strain DSM 11821 (NR_024697)	2						✓		10.36 ± 0.43	–	–	–	–	–
*Paenibacillus taichungensis* strain BCRC 17757 (NR_044428)	2							✓	–	–	–	–	–	–
*Lysinibacillus boronitolerans* strain 10a (NR_041276)	1		✓						26.69 ± 0.64	–	–	–	0.13 ± 0.35	–
*Lysinibacillus sphaericus* C3-41 (NR_074883)	1					✓			–	–	–	–	–	–
*Paenibacillus pabuli* strain HSCC 492T (NR_040853)	1							✓	16.36 ± 0.45	–	–	–	–	–

## Discussion

Quanzhou bay is reported to have varying degrees of heavy metal contaminations. Despite the adverse environmental impacts, *S. alterniflora* still thrives and occupies dominant niche. A worldwide investigation indicated that wetlands soil consist of Proteobacteria, Bacteroidetes, Acidobacteria, Firmicutes and Actinobacteria as the five major phyla^22^. Meanwhile, a previous study also shows that Proteobacteria, Bacteroidetes, Chloroflexi and Firmicutes are major phyla of rhizoplane bacteria in *S. alterniflora* monoculture^18^. Based on our study, Chloroflexi (47.20%) was the most abundant phylum in rhizosphere soil. Its relative abundance was higher than the Chloroflexi recovered from the other heavily contaminated sediment samples^23^. Chloroflexi is generally found in intertidal sediment and moderately acidic wetland^24,25^, and is believed to be associated with nitrite-oxidizing^26^, biological nutrient removal (BNR) processes^27^, sediment carbon cycling^28^, reductive dehalogenation of polychlorinated biphenyls and organohalide-respiring^29,30^, etc.

Approximately 9% of OTUs were allocated to six classes in Proteobacteria. Among them, the Delta-, gamma- and betaproteobacteria together accounted for 85.3% of proteobacterial OTUs. It is documented that Deltaproteobacteria is a major group of sulfur-reducing bacteria and the major class of phylum Proteobacteria in the rhizosphere of mangrove^3,31^. Endophytic roots of monoculture *S. alterniflora* are predominated by Proteobacteria, Cyanobacteria, Bacteroidetes, Firmicutes and Spirochaetes^18^. Our data show that although Actinobacteria is sensitive to Cd, Zn contamination^32^, it formed the largest phylum (39.27%) in endobacterial community. Actinobacteria have been studied as soil bacteria occur abundantly in most plants. They are thought to be indispensable in organic material decomposition, and were revealed as endophytes in endophytic roots of *S. alterniflora* in the present study. *Acidothermus* was the most frequently observed genus, followed by *Acidobacterium* (13.5%), *Demequina* (5.3%) and sulfate-reducing bacteria *Desulfovibrio*. Members in these genus are believed to play important roles in the metabolism of nitrogen, phosphorus, sulfur and some other organic compounds in wetland system^33^. Above all, both rhizo- and endobacterial community compositions were distinct from previous studies on that of *S. alterniflora* under normal growing environment^34,35^. These interesting differences observed in our study might be the results of the heavy metal stress on *S. alterniflora *in situ.

Site 3 was located at an oil terminal, the heavy metal content assessment revealed that rhizosphere soil collected from site 3 showed the highest concentration of Cr (see Supplementary Table S1 online). As shown in PCoA plot, both rhizo- and endobacterial data at site 3 were distinguished with those from other sites. Comprehensive comparison of bacterial potential ecological functions and their abundance in each site revealed a relative higher abundance of ecological functional bacteria at site 3 (see Supplementary Table S4 online). Previous studies demonstrated that high richness and abundance of sulfate-reducing bacteria (SRB) occurred in rhizosphere soil of *S. alterniflora* during the late growing season, suggesting that the abundant SRB might have close relationships with decomposition of soil organic matters produced by *S. alterniflora*^36^. Notably, microorganisms involved in sulfur cycle had significantly higher abundance in rhizosphere soil (1.48%) and root (11%) collected from site 3 compared to that collected from site 1 and 2. These sulfur cycle participants (mainly associated with sulfate-reduction) might also possess ability to oxidize acetate or other organic compounds^37^, and are considered as numerically important members on macrophyte root surfaces^38^. Those root-associated bacteria of *S. alterniflora* were great contributors to sulfur accumulation in *S. alterniflora*-invaded stands and could cause the higher sulfur concentration in situ than that of native plants or unvegetated zones^39^.

Other bacteria groups possess potential ecological functions such as phosphate solubilization, biodegradation, aromatic compound degradation, crude-oil degradation etc., shared the same pattern. Commonly, Anaerolineae is often recognized as a large component of microbial communities in sludge wastewater treatment plants^40^, and has been known to be associated with anaerobic degradation of oil-related compounds. Inconsistent with the previous reports, Anaerolineae was found constituted of over 18% of R3 but could barely found in R1 and R2. Interestingly, its abundance in rhizosphere soil appeared to be the lowest at site 3, indicating that *S. alterniflora* might accumulate bacteria that possess ecological functional to help its survival under various environment stresses.

*S. alterniflora* could enrich a certain amount of heavy metals. Screening of heavy metal resistant bacteria resulted in 16 different endophytes that showed resistance against at least one of the tested heavy metal ions. Some of the identified endophytes are previously studied as functional bacteria for phytoremediation. For example, a clone (38.82% of leave endophytes) showed resistance against three different heavy metal ions (Cu^2+^, Pb^2+^, Cr^6+^) was allocated to genus *Psychrobacter*, members of which are suggested to be applied in phytoextraction^41,42^. A *Lysinibacillus fusiformis* strain had the second abundant hits (14.29%), which is proved to have potentials for plant growth promotion due to their abilities to resist/reduce chromate at high level and resist/accumulate boron^43–45^.

Nitrogen (N_2_)-fixing bacteria are able to form symbiotic association with various plants^46^. This functional type of bacteria (e.g. *Azospirillum*, *Azotobacter*, *Oceanomonas*) have been isolated from rhizosphere and proved to have significant impact on the nitrogen cycle of the wetland ecosystem. Endophytic N_2_-fixing bacteria likely constitute only a small percentage of total endophytic bacteria, and the increase of the endophytic N_2_-fixing bacteria population has been considered as possible way for plants to increase nitrogen fixation^47,48^. Endophytic N_2_-fixing bacteria were found in previous researches: a diversity of *Azoarcus *spp. has been recovered from Kallar grass^49^; and *Klebsiella *sp. *strain Kp342* have been proved to fix N_2_ in wheat^50^. Likewise, N_2_-fixing endophytes seem to relieve nitrogen deficiencies of sweet potato in nitrogen-poor soils^51^. Moreover, some members of *Paenibacillus* are found to be N_2_-fixing bacteria^52^. In this case, we inoculated all endophytic isolations on non-nitrogen medium and obtained microbes from *Paenibacillus*, *Lysinibacillus*, and *Chryseobacterium*. *Paenibacillus* were identified in both leaves and roots, indicating that they might promote plant growth through fixing atmospheric nitrogen and dissolving phosphate.

Bacterial endophytes could promote plant growth by a number of different mechanisms, such as production of phytohormones^53^, phosphate solubilization activity^54,55^, nitrogen fixation^56^, siderophore biosynthesis^57,58^, and providing essential nutrients to host plants^59^. Like PGPR, endophytes can also promote plant growth by expressing ACC deaminase. In addition, the resistance of plants treated with ACC deaminase-containing PGPR to flood and heavy metal stress is significantly enhanced^60,61^. IAA, a plant hormone that does not apparently function as a hormone in bacterial cells, may have evolved in bacteria due to its importance in bacterium–plant relationship. In biochemical characterization of the isolated heavy metal-resistant clones, 13 IAA-producing isolates were identified. Among them, 5 isolates showed positive reaction in ACC-deaminase assay. The 16S rRNA sequencing results suggested that 5 ACC deaminase-containing endophytes were from *Psychrobacter*, *Lysinibacillus* and *Bacillus*. In addition, these isolates showed high levels of IAA synthesis. These results indicated that *S. alterniflora* was colonized by various kinds of endophytes. Some of the endophytes could tolerant a certain concentration of heavy metal and produce bacterial products to satisfy self-survival, improve plant tolerance to heavy metals and promote plant growth. To better understand how the rhizo- and endobacteria contribute to the heavy metal resistance of *S. alterniflora*, high-throughput sequencing assay focusing on functional genes is warranted for further study.

In conclusion, sediment of Quanzhou bay was contaminated with various degrees of heavy metals. This study contributes to our understanding of the composition and potential ecological functions of the rhizo- and endobacteria associated with *S. alterniflora*. The overall pattern of both rhizo- and endobacterial community structures were different from that reported in previous studies. The site 3 was located at an oil terminal with high level of Cr contamination. The rhizobacterial diversity decreased at site 3, root endophytes evolved to higher diversity with a large proportion of ecological functional bacteria for heavy metal accumulations, host plant growth promotion and crude-oil degradation. Some comments have suggested that *S. alterniflora* could alter the community structure of related functional microorganisms, even affect the carbon, nitrogen, and sulfur cycles in habitat^62,63^. Based on the analysis of datasets in the present study, root-associated bacteria of *S. alterniflora* might have the potential to affect nutrient metabolism in wetland ecosystem, especially nitrogen, phosphate, sulfur and carbon cycles. Culture-dependent method together with biochemical assay revealed that endophytes could tolerant certain concentration of heavy metals. Meanwhile, they could act through nitrogen-fixing, phosphate-solubilizing, IAA-producing and ACC-deaminase producing to participate in energy cycles and promote plant growth. Further investigation on the data indicated a considerable proportion of microorganisms with the potential to be applied in phytoremediation and natural medicine development. The functions of these microbial communities need to be further studied so as to elucidate the mechanism of *S. alterniflora* invasion and survival under heavy metal stress.

## Materials and methods

### Sample collection

Plants, rhizosphere soil and sediment samples were sampled from three sites of wetland in Quanzhou bay, Fujian, PR China in Dec 4, 2012. Sampling site 1 is located at a sluice of a residential quarter (N24°52.493′, E118°36.764′), site 2 was located at the north-west coast of Jinjiang bridge (N24°52.603′, E118°37.635′) and site 3 is at Houzhu oil terminal (N24°52.655′, E118°40.936′). All subsequent handling of samples was treated with sterilized gloves or tools. Plants with rhizosphere soil were immediately sealed in polyethylene bags and transported to the laboratory in transportable cooler (4 °C). Fresh samples were sorted as rhizosphere soil (S), roots (R) and leaves (L).

### Sample pretreatments

Rhizosphere soil (S) was carefully removed from the root of the *S. alterniflora*, then stored at 4 °C. Plant samples were surface sterilized as Idris described^64^. Sterility was checked by blotting plant surface tightly onto tryptic soy agar (TSA) plates and incubating plates at 28 °C for 2 days. Surface-sterilized plant samples were cut into small pieces and classified into leaves (L) and roots (R), then stored at 4 °C.

### Screening of heavy metal resistant microorganisms

After pretreatment, fresh rhizosphere soil (2.5 g) and sterilized plant samples (R and L) were soaked in tryptic soy broth and shaken for 2 h at 250 rpm at 28 °C, respectively. The solution was then left without shaking for 1 h to allow the settlement of particles. Various tenfold dilutions were plated on TSA (with 100 mg L^−1^ cycloheximide), nitrogen-free culture medium and TSA contain different concentrations of CrCl_6_ (5–50 ppm), CdCl_2_ (2–10 ppm), Pb(NO_3_)_2_ (20–100 ppm), ZnCl_2_ (100–500 ppm), CuSO_4_ (20–100 ppm), NiSO_4_ (10–50 ppm), respectively. Plates were incubated at 28 °C until visible clones were observed.

### DNA extraction

TIANamp Bacteria DNA kit (TIANGEN) was applied for DNA extraction from clones. For environmental DNA extraction, rhizosphere soil (S), root (R) and leaves (L) were grinded using liquid nitrogen respectively, Soil DNA Kit (Sigma) and Plant DNA Kit (Sigma) were applied to acquire high-quality DNA from rhizosphere soil and plant samples. Equal amount of DNA isolated from 6 samples were pooled into one for further experiments (e.g. the leaf DNA contained the equal amount of DNA that isolated from 6 individual plants). Isolated DNA was resuspended in 50–200 μL sterile water and kept at − 80 °C until use.

### Full-length and segment 16S rRNA sequencing

The 16S rRNA region of environmental DNA and DNA extraction from clones was amplified (primers 20F-1503R for rhizobacteria, while 773F-1513R for endophytes to avoid affection of chloroplast)^65^. PCR products were checked on 1.5% Sepharose gel. Amplified fragments were recovered by QIAquick Gel Extraction Kit (QIAGEN) and inserted into pMD18-T vector, then transformed into *E. coli* (DH5α) component cells. Colonies (~ 50) were randomly picked for sanger sequencing. The results were subjected to NCBI blast analysis to see whether the results obtained from sanger sequencing is a subset of the 454-sequencing results.

### 454 Pyrosequencing of V4 16S rRNA tags

Environmental DNA extraction of root and rhizosphere soil were obtained as described above, subsequently quantified with Qubit 2.0 DNA Kit (Invitrogen) and checked for integrity and concentration. High-quality DNA was then proceeded to PCR with barcode-infused universal primers (see Supplementary Table S5 online). PCR products were purified and quantified by Qubit 2.0 DNA Kit. All parallel samples were balanced mixed and sequenced at Sangon, using GS FLX titanium system.

### Data analysis

Differentiating sequences by their own barcodes, low complexity sequences were ruled out by Prinseq (version 0.20.4, https://prinseq.sourceforge.net/). To obtain high quality reads, we applied Lucy (1.20p, https://lucy.sourceforge.net/) to trim the inferior quality (Q < 20) domain in both ends of the sequences and reserved sequences with length no less than 50 bp using sliding window method. Potential chimeras were identified and removed using ChimeraSlayer^66^. Ribosomal Database Project Classifier (RDP v2.2, https://rdp.cme.msu.edu/classifier/classifier.jsp) was applied to classify sequences. Sequences were clustered according to the distance between them, and were subsequently distributed into different operational taxonomic units (OTUs) at 97% similarity using QIIME Software (v1.80, https://qiime.org/). Alpha and beta diversity estimates were calculated by Mothur Software (mothur v1.31.2: https://www.mothur.org/). Bacterial diversity was estimated with N_obs_ (observed richness), Shannon Weiner diversity index (H’), Simpson index (D), evenness (E) ChaoI, Margalef index (d_Ma_), Menhinick index (R_2_), Pielou index (J_gi_), coverage and PIE after trimming each sample to an equal number of tags^67–69^. Algorithm Unifrac was performed to calculate sample intervals, clustering and principal component analysis (PCA). Figure was drawn using R (v3.1.1, https://www.datavis.ca/R/). Based on the results from RDP classifier (v2.2), relative abundance of each rank at different levels were calculated and compared to find the microflora that showed significant variation between samples (Fisher’s exact test) or groups (T statistic permutation test).

### Phosphate solubilization assay

After sequencing, endophyte clones obtained from TSA (containing heavy metal) were preserved and preceded to following tests. Endophyte clones were inoculated to inorganic phosphorus medium, plates were incubated for 5 days at 28 °C. Colonies with soluble phosphorus circles were considered as phosphate-solubilizing strains.

### IAA analysis

l-Tryptophan (2.5 mg mL^−1^) was filter sterilized before use. Medium containing 4 mL of nitrogen medium and 1 mL of l-tryptophan (2.5 mg mL^−1^) was inoculated with endophyte clone, and shaken for 4 days at 250 rpm at 28 °C. Removed 1 mL of each suspension and mixed thoroughly with 2 mL Sackowski’s reagent^70^, tubes were shielded from light at room temperature for 30 min. Positive reactions with IAA production showed pink color. Standard curve was established with various tenfold dilutions of IAA standard solution measured at 530 nm for absorbance. Endophyte clones were cultured in nitrogen medium with l-tryptophan (0.5 mg mL^−1^) for 48 h before measured for absorbance at 600 nm. Supernatants were mixed with isovolumetric Sackowski’s reagent, developed in dark for 30 min, and subjected to measurement for absorbance at 530 nm. Data were recorded for further analysis.

### ACC-deaminase production analysis

Endophyte clones were inoculated on DF salts minimal medium respectively^71^, and incubated at 30 °C. Clones that could grow and pass for 5 times were considered able to produce ACC-deaminase. Positive clones were inoculated in 15 mL TSB, shaken for 24 h at 28 °C. Cells were harvested by centrifugation for 10 min at 8000×*g* at 4 °C, rinsed with DF salts medium for three times. Cells were resuspended with 7.5 mL ADF medium (DF salts medium with ACC final concentration of 3.0 mM), shaken for 24 h at 200 rpm at 30 °C to induce the production of ACC-deaminase. Cells were harvested by centrifugation for 10 min at 8000×*g* at 4 °C, rinsed with 0.1 M Tris–HCl (pH 7.6) twice. Samples were resuspended in 600 μL 0.1 M Tris–HCl (pH 8.5), added 30 μL methylbenzene and vortexed for 30 s to make crude enzyme. Protein concentration was determined by Bradford protein assay kit (Thermo Fisher) following the manufacturer’s instruction. Samples were subjected to ACC-deaminase activity test. Briefly, mixed 200 μL crude enzyme and 20 μL ACC (0.5 M), incubated at 30 °C for 15 min, added 1 mL 0.56 M HCL to terminate the reaction (reaction without crude enzyme and reaction without ACC were set up as control groups). Supernatant (1 mL) was removed to a new tube and mixed with 800 μL HCl (0.56 M) and 300 μL 0.2% 2,4-dinitrophenyl hydrazine (dissolved in 2 M HCl). After incubation at 30 °C for 30 min, samples were mixed thoroughly with 2 mL 2 M NaOH, and measured for the absorbance at 540 nm. Α-ketobutyrate standard curve was set up as followed: dissolved 0.102 g α-ketobutyrate in 10 mL 0.1 M Tris–HCl (pH 8.5) to obtain a stocking solution of 100 mM α-ketobutyrate. The stocking solution was diluted into 10 mM before use. Removed 0, 10, 20, 40, 60, 80, 100, 120 μL 10 mM dilution to tubes respectively and added 0.1 M Tris–HCl (pH 8.5) to final volume of 1 mL. The final concentration range of α-ketobutyrate was 0.024–0.293 μM. Samples were processed as described above to establish a standard curve. ACC-deaminase activity was defined by the μmol of α-ketobutyrate produced by 1 mg enzyme protein in the reaction per hour, enzyme-activity unit was α-ketobutyrate μmol/(mg·h).

### Siderophore production analysis

Endophyte clones were inoculated on CAS agar plates^72^. Plates were incubated for 8–12 days at 30 °C. Colonies with yellow halos were considered as able to produce siderophore on CAS agar plate. To quantify siderophore production, solutions and procedures were carried out as previous described^72^. The quantitative index defined as As/Ar: 0–0.2, +++++; 0.2–0.4, ++++; 0.4–0.6, +++; 0.6–0.8, ++; 0.8–1.0, + Siderophore units were defined as below:

$$ {\raise0.7ex\hbox{${({\text{As}} - {\text{Ar}})}$} \!\mathord{\left/ {\vphantom {{({\text{As}} - {\text{Ar}})} {{\text{Ar}}}}}\right.\kern-\nulldelimiterspace} \!\lower0.7ex\hbox{${{\text{Ar}}}$}} \times 100 = \% \;{\text{siderophore}}\;{\text{ units}} $$

### Heavy metal content analysis

Sediments and rhizosphere soil samples were determined using microwave digestion-ICP-MS following the prior description^73^.

## Supplementary information

Supplementary Information.
