# Supplementary material for: Characterization of bacterial communities associated with the exotic and heavy metal tolerant wetland plant Spartina alterniflora
Source: Sci Rep. 2020 Oct 22;10:17985. doi: 10.1038/s41598-020-75041-5 (PMC7583234; doi:10.1038/s41598-020-75041-5)
Supplement: Supplementary file 1 — Supplementary Information. [file 41598_2020_75041_MOESM1_ESM.pdf]

Supplementary information
for
Characterization of bacterial communities associated with the exotic and heavy metal tolerant wetland plant *Spartina alterniflora*

Ying Yang[1,2*], Jian Ding[2,3*], Yulang Chi[1], Jianjun Yuan[1]

[1]College of Oceanology and Food Science, Quanzhou Normal University, Quanzhou 362000, China

[2]State Key Laboratory of Marine Environmental Science, College of Ocean and Earth Sciences, Xiamen University, Xiamen 361005, China

[3]Sunshine Guojian Pharmaceutical (Shanghai) Co., Ltd

Corresponding authors: ylchi@qztc.edu.cn (Y.C), yuanjianjun2005@qztc.edu.cn (J.Y)

[*]These authors contributed equally to this work.

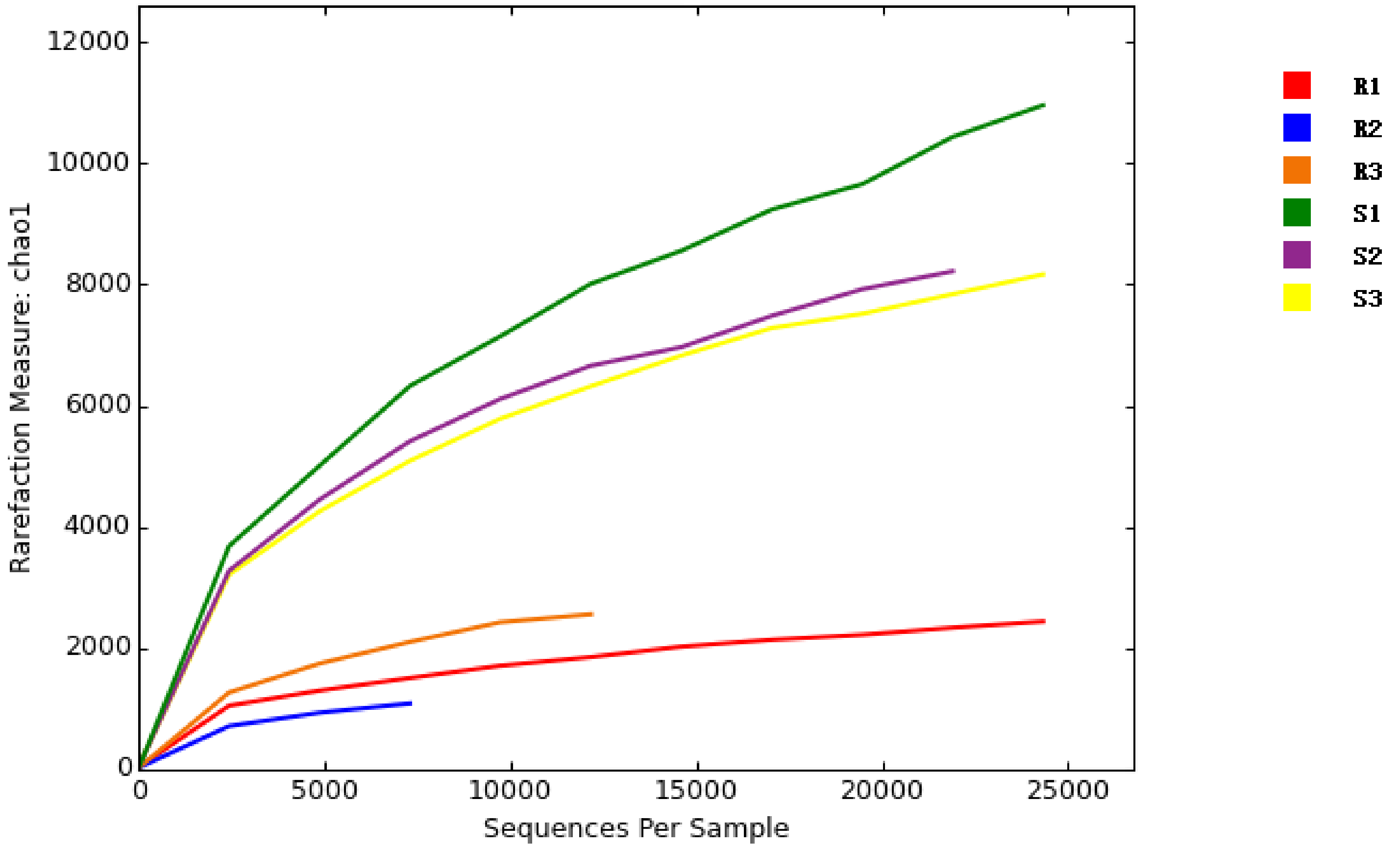

Figure S1. Rarefaction curves for samples.

Abbreviations: R, roots of *Spartina alterniflora*; S, rhizosphere soil of *S. alterniflora*. Figure was drawn using R (v3.1.1, http://www.datavis.ca/R/).

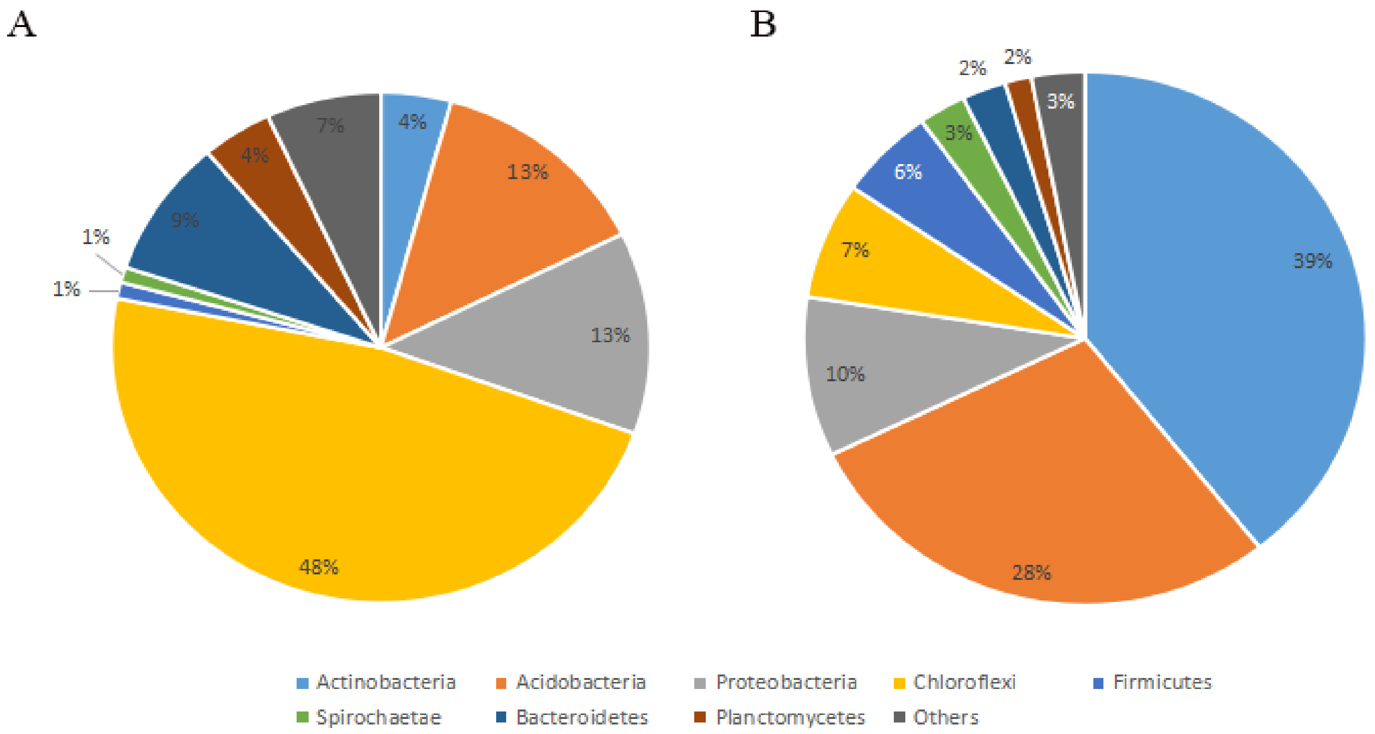

Figure S2. Phylum level taxonomic and relative abundance for (A) rhizo- and (B) endobacterial communities.

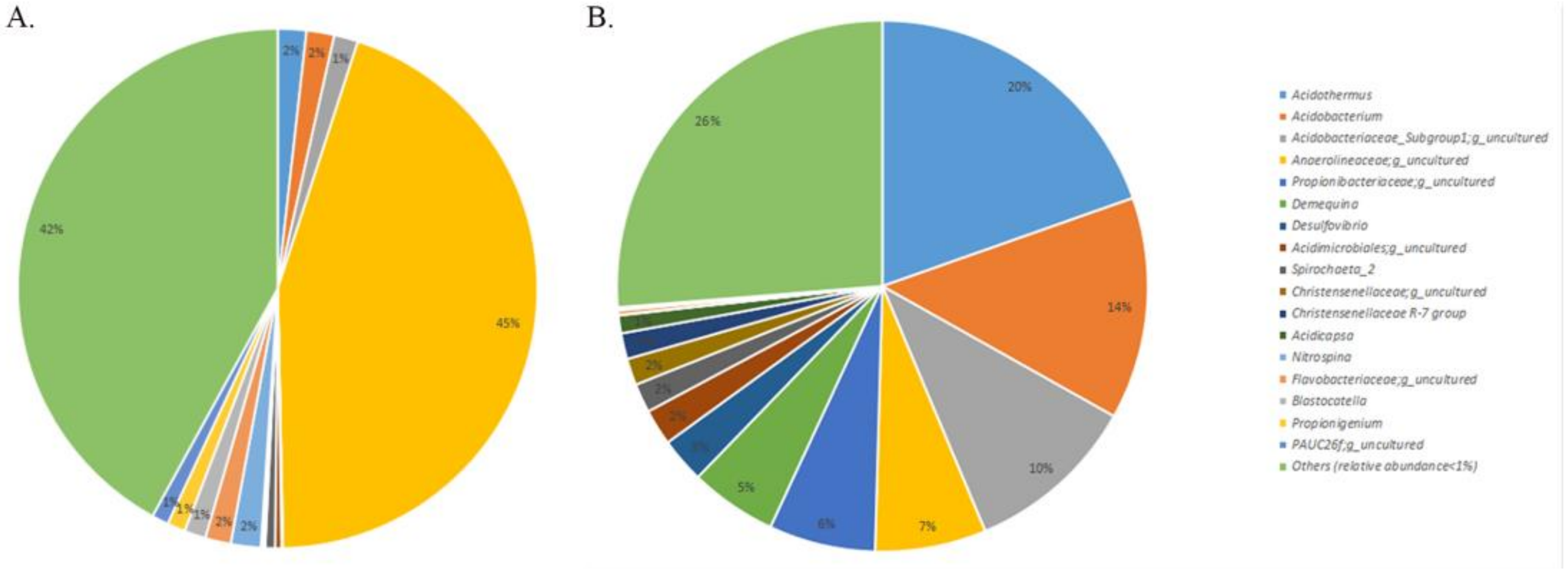

Figure S3. Genus level taxonomic and relative abundance for (A) rhizo- and (B) endobacterial communities.

Table S1. Heavy metal content in sediments and rhizosphere soil of Quanzhou Bay

Element	Sediment			Rhizosphere soil		
	1	2	3	1	2	3
Cr/μg g^{-1}	319.64	312.94	284.47	175.67	130.33	258.33
Ni/μg g^{-1}	78.51	95.96	84.43	111.33	76.67	169.00
Cu/μg g^{-1}	178.81	159.71	140.39	42.00	40.67	31.33
Zn/μg g^{-1}	692.29	732.37	599.04	220.67	189.00	158.00
Cd/μg g^{-1}	3.73	3.09	2.67	6.00	3.33	6.00
Pb/μg g^{-1}	221.09	200.29	202.39	119.33	85.33	95.33

Table S2. Potential ecological function of non-culturable clones sequencing results for which obvious metabolisms can be inferred

Bacterial/ Archaeal Taxonomy	Potential ecological function	Isolated from
Proteobacteria;Alphaproteobacteria;Caulobacterales;Caulobacteraceae;Brevundimonas	Denitrification	Soil, root, leaf
Proteobacteria;Alphaproteobacteria;Pseudomonadales;Moraxellaceae;Acinetobacter	Denitrification	Soil, leaf
Proteobacteria;Gammaproteobacteria;Aeromonadales;Aeromonadaceae;Aeromonas	Gas production<br>Protein-dissolving	Root, leaf
Proteobacteria;Alphaproteobacteria;Rhizobiales;Xanthobacteraceae;Xanthobacter	Dinitrogen fixation	Root, leaf
Proteobacteria;Alphaproteobacteria;Rhizobiales;Hypomicrobiaceae;Devosia	Nitrogen fixation,nitrification<br>Crude-oil tolerant<br>Hexachlorocyclohexane tolerant	Root, leaf
Proteobacteria;Gammaproteobacteria;Oceanospirillales;Oceanospirillaceae;Marinomonas	Heavy metal-tolerant	Root, leaf
Proteobacteria;Alphaproteobacteria;Rhizobiales;Hypomicrobiaceae;Aquabacter	Gas-vacuolate	Root, leaf
Proteobacteria;Alphaproteobacteria;Caulobacterales;Caulobacteraceae;Asticcacaulis		Root, leaf
Proteobacteria;Gammaproteobacteria;Alteromonadales;Shewanellaceae;Shewanella	Biodegradation<br>Denitrification	Root, leaf

Bacterial/ Archaeal Taxonomy	Potential ecological function	Isolated from
Firmicutes;Clostridia;Clostridiales;Clostridiales;Clostridium	Fatty-acid production<br>Dissimilatory Fe(III) reduction<br>Amino acid-oxidizing, acetate-reducing<br>Nitrogen fixation,ammonia-producing<br>Biodegradation<br>Benzaldehyde-converting<br>Cr(VI) resistant	Soil
Firmicutes;Bacilli;Bacillales;Bacillales Family XII. Incertae Sedis;Exiguobacteirum	Catalase activity	Soil
Pelobacteraceae;Planctomycetia;Planctomycetales;Planctomycetaceae;Pirellula		Soil
Planctomycetes;Planctomycetia;Planctomycetales;Planctomycetaceae;Rhodopirellula		Soil
Proteobacteria;Deltaproteobacteria;Desulfuromonadales;Pelobacteraceae;Pelobacter	Trihydroxybenzenes fermentation<br>Hydroxyhydroquinone degradation<br>Selenate respiration	Soil
Unclassified Bacteria;candidate division NC10;Candidatus Methylomirabilis	Nitrite-driven anaerobic methane oxidation	Soil
Cyanobacteria;Oscillatoriophycideae;Oscillatoriales;Oscillatoriales Family Incertae Sedis;Trichodesmium		Soil
Proteobacteria;Gammaproteobacteria;Enterobacteriales;Enterobacteriaceae;Pantoea		Root
Proteobacteria;Alphaproteobacteria;Rhodospirillales;Rhodospirillaceae;Azospirillum	Nitrogen fixation<br>Sulfur-loving	Root
Bacteroidetes;Cytophagia;Cytophagales;Flammeovirgaceae;Marivirga		Root
Archaea;Euryarchaeota;Methanomicrobia;Methanomicrobiales;Methanomicrobiaceae;	Methanogenic	Root

**Bacterial/ Archaeal Taxonomy**	**Potential ecological function**	**Isolated from**
Methanoplanus		
Proteobacteria;Alphaproteobacteria;Rhodobacterales;Rhodobacteraceae;Nautella		Root
Firmicutes;Bacilli;Bacillales;Bacillaceae;Bacillus	Biodegradation	Root
	As/Te/Se-resistant,As/Se-reduction	
	Fe(III)- and	
	Mn(IV)-reducing,Mn-oxidizing	
	Nitrogen fixation	
	Thiosulfate oxidation	
	Plant-growth-promoting	
	Diesel oil degradation	
	Antibacterial activity,	
Proteobacteria;Alphaproteobacteria;Caulobacterales;Caulobacteraceae;Caulobacter		Leaf
Proteobacteria;Alphaproteobacteria;Rhodobacterales;Rhodobacteraceae;Labrenzia		Leaf
Bacteria;Proteobacteria;Betaproteobacteria;Burkholderiales;Comamonadaceae;Leptothrix		Leaf
Proteobacteria;Alphaproteobacteria;Rhizobiales;Phyllobacteriaceae;Mesorhizobium	Nitrogen fixation	Leaf
	Cellulose degradation	
	Metal resistant	
	Sulfur oxidation	
Proteobacteria;Alphaproteobacteria;Rhizobiales;Rhizobiaceae;Agrobacterium		Leaf
Proteobacteria;Alphaproteobacteria;Caulobacterales;Caulobacteraceae;Phenylobacterium	Herbicide chloridazon degradation	Leaf
Proteobacteria;Gammaproteobacteria;Pseudomonadales;Pseudomonadaceae;Pseudomonas	Antagonistic to plant pathogens	Leaf
	Arsenite oxidation	
	Nitrogen fixation	
	Nitrification	

Bacterial/ Archaeal Taxonomy	Potential ecological function	Isolated from
	Denitrification	
	Biodegradation	
Proteobacteria;Gammaproteobacteria;Chromatiales;Chromatiaceae;Rheinheimera	Antimicrobial acitivity	Leaf
Proteobacteria;Gammaproteobacteria;Enterobacteriales;Enterobacteriaceae;Serrati		Leaf
Proteobacteria;Alphaproteobacteria;Rhizobiales;Rhizobiaceae;Sinorhizobium	Growth-promoting	Leaf
	Resistant to multiple antibiotics	
Proteobacteria;Alphaproteobacteria;Rhodospirillales;Acetobacteraceae;Stella		Leaf
Proteobacteria;Gammaproteobacteria;Enterobacteriales;Enterobacteriaceae;Yokenella		Leaf

Table S3. Potential ecological function of culturable clones sequencing results for which obvious metabolisms can be inferred

Bacterial/ Archaeal Taxonomy	Potential ecological function	Isolated from
Firmicutes;Bacilli;Bacillales;Bacillaceae;Bacillus	Biodegradation	Soil, root, leaf
	As/Te/Se-resistant,As/Se-reduction	
	Fe(III)- and	
	Mn(IV)-reducing,Mn-oxidizing	
	Nitrogen fixation	
	Thiosulfate oxidation	
	Plant-growth-promoting	
	Diesel oil degradation	
	Antibacterial activity,	
Flavobacteria;Flavobacteriales;Flavobacteriaceae;Chryseobacterium	Amoebae-resisting	Soil, root, leaf
Proteobacteria;Alphaproteobacteria;Pseudomonadales;Moraxellaceae;Acinetobacter	Denitrification	Soil, root
Proteobacteria;Gammaproteobacteria;Aeromonadales;Aeromonadaceae;Aeromonas	Gas production	Soil, root
	Protein-dissolving	
Bacteroidetes;Flavobacteria;Flavobacteriales;Flavobacteriaeceae;Cloacibacterium		Soil
Firmicutes;Bacilli;Bacillales;Bacillales Family XII. Incertae Sedis;Exiguobacteirium	Catalase activity	Soil
Firmicutes;Bacilli;Bacillales;Bacillaceae;Halobacillus		Soil
Bacteroidetes;Flavobacteriia;Flavobacteriales;Flavobacteriaceae;Flavobacterium	Arsenic resistant	Soil
	Arsenite oxidation	
	Hexachlorocyclohexane resistant	
	Denitrification	
	Biodegradation	
Proteobacteria;Gammaproteobacteria;Enterobacteriales;Enterobacteriaceae;Enterobacte	Nitrogen fixation	Root

Bacterial/ Archaeal Taxonomy	Potential ecological function	Isolated from
r		
Firmicutes;Bacilli;Bacillales;Planococcaceae;Planococcus	Hydrocarbon degradation<br>Biodegradation	Root
Proteobacteria;Gammaproteobacteria;Pseudomonadales;Pseudomonadaceae;Pseudomonas	Antagonistic to plant pathogens<br>Arsenite oxidation<br>Nitrogen fixation<br>Nitrification<br>Denitrification<br>Biodegradation	Root
Firmicutes;Bacilli;Bacillales;Paenibacillaceae;Paenibacillus	Biodegradation<br>Nitrogen fixation<br>Antimicrobial activity<br>PAH degradation<br>Ammonification<br>Denitrification	Leaf
Actinobacteria;Actinobacteria;Corynebacteriales;uncultured;uncultured Microbacterium	Biodegradation<br>Crude-oil degradation<br>Hexachlorocyclohexane resistant	Leaf

Table S4. Potential ecological function of tag sequences for which obvious metabolisms can be inferred

Potential ecological function	Bacterial/ Archaeal Taxonomy	Relative abundance (%)					
**Bacteria**		R1	R2	R3	S1	S2	S3
Desulfurization	*Proteobacteria;Deltaproteobacteria;Desulfobacterales;Desulfobacteraceae;Desulfobacterium*	0	0	0	0	0	7.29e-03
Dibenzothiophene desulphurization	*Actinobacteria;Actinobacteria;Corynebacteriales;Nocardiaceae;Gordonia*	0	1.08e-02	0	0	0	7.29e-03
Dimethylsulfide oxidation	*Proteobacteria;Gammaproteobacteria;Thiotrichales;Piscirickettsiaceae; Methylophaga*	1.19e-02	1.08e-02	0	2.42e-02	4.25e-03	1.09e-02
Dissimilatory sulfate reduction	*Proteobacteria;Deltaproteobacteria;Desulfobacterales;Desulfobacteraceae;Desulfococcus*	0	0	1.33	1.61e-02	0	1.09e-02
	Proteobacteria;Deltaproteobacteria;Desulfobacterales;Desulfobacteraceae;Desulfonema	0	0	6.86e-03	0	0	2.19e-02
	Proteobacteria;Deltaproteobacteria;Desulfobacterales;Desulfobacteraceae;Desulfosarcina	0	0	6.86e-03	5.37e-03	8.49e-03	4.74e-02
	Proteobacteria;Deltaproteobacteria;Desulfobacterales;Desulfobacteraceae;Desulfotignum	0	0	0	5.37e-03	0	3.65e-03
	Proteobacteria;Deltaproteobacteria;Desulfobacterales;Desulfobulbaceae; Desulfobulbus	3.98e-03	0	8.91e-02	1.77e-01	1.53e-01	4.45e-01
	Proteobacteria;Deltaproteobacteria;Desulfobacterales;Desulfobacteraceae;Desulfobacter	7.96e-03	3.25e-02	4.11e-02	2.69e-03	0	7.29e-03
Sulfate reduction	*Proteobacteria;Deltaproteobacteria;Desulfarculales;Desulfarculaceae;*	2.39e-02	0	0	2.95e-02	2.55e-02	4.74e-02

Potential ecological function	Bacterial/ Archaeal Taxonomy	Relative abundance (%)					
	Desulfatiglans						
	Proteobacteria;Deltaproteobacteria;Desulfobacterales;Desulfobacteraceae;Desulfatibacillum	0	0	0	5.37e-03	0	7.29e-03
	Proteobacteria;Deltaproteobacteria;Desulfobacterales;Desulfobacteraceae;Desulfatiferula	0	0	0	2.69e-03	0	7.29e-03
	Proteobacteria;Deltaproteobacteria;Desulfobacterales;Desulfobacteraceae;Desulfatitalea	0	0	0	0	0	3.65e-03
	Proteobacteria;Deltaproteobacteria;Desulfobacterales;Desulfobacteraceae;Desulfobacula	0	0	6.17e-02	0	1.70e-02	4.74e-02
	Proteobacteria;Deltaproteobacteria;Desulfobacterales;Desulfobacteraceae;Desulfofaba	0	0	0	0	0	3.65e-03
	Proteobacteria;Deltaproteobacteria;Desulfobacterales;Desulfobacteraceae;Desulfofrigus	0	0	0	0	0	3.65e-03
	Proteobacteria;Deltaproteobacteria;Desulfobacterales;Desulfobacteraceae;Desulfospira	0	0	0	2.69e-03	0	1.46e-02
	Proteobacteria;Deltaproteobacteria;Desulfobacterales;Desulfobulbaceae; Desulfocapsa	0	0	1.37e-02	2.69e-03	1.27e-02	2.19e-02
	Proteobacteria;Deltaproteobacteria;Desulfobacterales;Desulfobulbaceae; Desulfopila	3.98e-03	0	2.13e-01	1.34e-02	1.27e-02	1.09e-02
	Proteobacteria;Deltaproteobacteria;Desulfobacterales;Desulfobulbaceae; Desulforhopalus	1.59e-02	6.50e-02	7.54e-02	2.69e-03	0	1.82e-02
	Proteobacteria;Deltaproteobacteria;Desulfobacterales;Desulfobulbaceae;	0	5.42e-02	6.86e-02	5.37e-03	8.49e-03	2.19e-02

**Potential ecological function**	**Bacterial/ Archaeal Taxonomy**	**Relative abundance (%)**					
	Desulfotalea						
	Proteobacteria;Deltaproteobacteria;Desulfovibrionales; Desulfovibrionaceae;Desulfovibrio	2.55e-01	5.20e-01	7.50	5.37e-03	8.49e-03	4.01e-02
	Proteobacteria;Deltaproteobacteria;Syntrophobacterales;Syntrophaceae; Desulfobacca	0	0	0	2.69e-03	4.25e-03	0
	Proteobacteria;Deltaproteobacteria;Syntrophobacterales;Syntrophaceae; Desulfomonile	0	0	2.74e-02	2.69e-03	4.25e-03	1.09e-02
	Proteobacteria;Deltaproteobacteria;Syntrophobacterales; Syntrophobacteraceae;Desulfovirga	0	0	0	0	8.49e-03	0
	Proteobacteria;Deltaproteobacteria;Syntrophobacterales; Syntrophobacteraceae;Syntrophobacter	0	0	0	8.06e-03	4.25e-03	0
Sulfur oxidation	*Proteobacteria;Alphaproteobacteria;Rhizobiales;Phyllobacteriaceae; Mesorhizobium*	3.98e-03	0	2.74e-02	0	0	0
	Proteobacteria;Alphaproteobacteria;Rhodobacterales;Rhodobacteraceae; Citreicella	0	0	0	2.69e-03	0	0
	Proteobacteria;Alphaproteobacteria;Rhodospirillales;Acetobacteraceae; Acidicaldus	1.59e-02	1.08e-02	1.37e-02	0	0	3.65e-03
	Proteobacteria;Betaproteobacteria;Rhodocyclales;Rhodocyclaceae; Sulfuritalea	0	0	0	2.15e-02	1.70e-02	0
	Proteobacteria;Epsilonproteobacteria;Campylobacterales; Helicobacteraceae;Sulfuricurvum	0	0	0	1.32e-01	4.25e-03	7.29e-03
	Proteobacteria;Gammaproteobacteria;Chromatiales;Chromatiaceae;	0	0	0	5.37e-03	0	0

**Potential ecological function**	**Bacterial/ Archaeal Taxonomy**	**Relative abundance (%)**					
	Thiohalobacter						
	Proteobacteria;Gammaproteobacteria;Chromatiales; Ectothiorhodospiraceae;Thioalkalispira	0	0	6.86e-03	4.30e-02	8.49e-03	3.65e-02
	Proteobacteria;Gammaproteobacteria;Chromatiales; Ectothiorhodospiraceae;Thioalkalivibrio	0	0	0	0	0	3.65e-03
	Proteobacteria;Gammaproteobacteria;Chromatiales; Ectothiorhodospiraceae;Thiogranum	0	0	0	1.42e-01	5.09e-02	1.46e-02
	Proteobacteria;Gammaproteobacteria; Gammaproteobacteria_Incertae_Sedis;Unknown_Family;Sedimenticola	0	0	0	2.15e-02	1.27e-02	4.38e-02
	Proteobacteria;Gammaproteobacteria; Gammaproteobacteria_Incertae_Sedis;Unknown_Family;Thiohalophilus	0	0	0	5.10e-02	1.70e-02	2.19e-02
	Proteobacteria;Gammaproteobacteria; Gammaproteobacteria_Incertae_Sedis;Unknown_Family;Thiohalorhabdus	0	0	1.37e-02	0	0	3.65e-03
	Proteobacteria;Betaproteobacteria;Hydrogenophilales;Hydrogenophilaceae;Thiobacillus	0	0	0	3.76e-02	4.25e-03	0
	Proteobacteria;Epsilonproteobacteria;Campylobacterales; Helicobacteraceae;Sulfurimonas	7.96e-03	0	0	1.34e-02	1.70e-02	7.66e-02
	Proteobacteria;Gammaproteobacteria;Thiotrichales;Piscirickettsiaceae; Thiomicrospira	0	0	0	2.69e-03	0	0
Sulfur reduction	*Proteobacteria;Deltaproteobacteria;Desulfuromonadales; Desulfuromonadaceae;Desulfuromonas*	0	0	0	2.95e-02	8.07e-02	1.82e-02
	Proteobacteria;Deltaproteobacteria;Desulfuromonadales;	1.19e-02	0	6.86e-03	0	4.25e-03	2.92e-02

Potential ecological function	Bacterial/ Archaeal Taxonomy	Relative abundance (%)					
	Desulfuromonadaceae;Desulfuromusa						
	Proteobacteria;Epsilonproteobacteria;Campylobacterales; Campylobacteraceae;Sulfurospirillum	2.03e-01	0	2.74e-02	5.37e-03	0	7.29e-03
	Deferribacteres;Deferribacteres;Deferribacterales;Deferribacteraceae; Deferribacter	0	0	9.60e-02	0	0	0
	Deferribacteres;Deferribacteres;Deferribacterales;Deferribacteraceae; Geovibrio	0	0	0	0	0	3.65e-03
Thiosulfate reduction	*Firmicutes;Clostridia;Clostridiales;Family_XII;Fusibacter*	4.22e-01	4.12e-01	2.61e-01	4.30e-02	1.27e-01	1.68e-01
	Synergistetes;Synergistia;Synergistales;Synergistaceae;Dethiosulfovibrio	0	0	2.06e-02	0	0	0
	Proteobacteria;Epsilonproteobacteria;Campylobacterales; Helicobacteraceae;Sulfurovum	0	1.08e-02	1.05	4.30e-02	3.82e-02	2.22e-01
Thiosulfate oxidation	*Proteobacteria;Alphaproteobacteria;Rhizobiales;Bradyrhizobiaceae; Bosea*	7.96e-03	3.25e-02	1.37e-02	0	0	0
	Proteobacteria;Betaproteobacteria;Burkholderiales;Burkholderiaceae; Limnobacter	0	0	0	5.37e-03	0	0
Sum of S oxidation and sulfate reduction		1	1.16	10.97	0.91	0.65	1.48
Ammonia production	*Actinobacteria;Actinobacteria;Micrococcales;Microbacteriaceae; Curtobacterium*	1.43e-01	6.50e-02	2.06e-01	0	2.55e-02	5.47e-02
Ammonium oxidation	*Planctomycetes;Planctomycetacia;Brocadiales;Brocadiaceae; Candidatus_Scalindua*	0	0	0	8.06e-03	0	0
	Proteobacteria;Betaproteobacteria;Nitrosomonadales;Nitrosomonadaceae;	0	0	0	1.02e-01	8.49e-03	2.55e-02

Potential ecological function	Bacterial/ Archaeal Taxonomy	Relative abundance (%)					
	Nitrosomonas						
Denitrification	*Proteobacteria;Alphaproteobacteria;Rhizobiales;Hyphomicrobiaceae; Hyphomicrobium*	0	0	0	2.69e-03	4.25e-03	0
	Proteobacteria;Betaproteobacteria;Methylophilales;Methylophilaceae;Methylotenera	5.77e-01	2.49e-01	8.43e-01	7.04e-01	1.81	3.25e-01
	Proteobacteria;Alphaproteobacteria;Rhodospirillales;Rhodospirillaceae; Nisaea	0	0	6.86e-03	0	0	0
	Proteobacteria;Betaproteobacteria;Burkholderiales;Comamonadaceae； Comamonas	3.98e-03	0	6.86e-03	2.69e-03	8.49e-03	1.09e-02
	Proteobacteria;Betaproteobacteria;Burkholderiales;Comamonadaceae; Variovorax	7.96e-03	0	0	0	4.25e-03	0
	Proteobacteria;Betaproteobacteria;Rhodocyclales;Rhodocyclaceae; Dechloromonas	3.98e-03	0	0	2.95e-02	3.82e-02	0
	Proteobacteria;Betaproteobacteria;Rhodocyclales;Rhodocyclaceae; Denitratisoma	0	0	0	5.37e-03	0	0
	Proteobacteria;Betaproteobacteria;Rhodocyclales;Rhodocyclaceae;Thauera	1.19e-02	0	0	1.07e-02	4.25e-02	1.82e-02
	Proteobacteria;Betaproteobacteria;Rhodocyclales;Rhodocyclaceae;Zoogloea	0	0	0	8.06e-03	2.97e-02	3.65e-03
	Proteobacteria;Gammaproteobacteria;Aeromonadales;Aeromonadaceae; Zobellella	0	0	0	0	4.25e-03	0
	Proteobacteria;Gammaproteobacteria;Alteromonadales;Shewanellaceae; Shewanella	7.96e-03	1.08e-02	0	8.06e-02	1.91e-01	4.74e-02
	Proteobacteria;Gammaproteobacteria;Gammaproteobacteria_Incertae_Sedis;	0	0	1.37e-02	0	0	3.65e-03

Potential ecological function	Bacterial/ Archaeal Taxonomy	Relative abundance (%)					
	Unknown_Family;Thiohalorhabdus						
	Proteobacteria;Gammaproteobacteria;Oceanospirillales;Halomonadaceae; Halomonas	0	0	0	8.06e-03	0	0
	Proteobacteria;Gammaproteobacteria;Xanthomonadales; Xanthomonadales_Incertae_Sedis;Steroidobacter	0	0	0	0	1.70e-02	0
Nitrate reduction	*Deferribacteres;Deferribacteres;Deferribacterales;Deferribacteraceae; Denitrovibrio*	7.17e-02	2.71e-01	9.60e-02	0	0	1.46e-02
	Actinobacteria;Actinobacteria;Propionibacteriales;Nocardioidaceae; Nocardioides	1.59e-02	2.17e-02	6.17e-02	8.06e-03	1.70e-02	5.83e-02
	Bacteroidetes;Bacteroidia;Bacteroidia_Incertae_Sedis;Prolixibacteraceae; Prolixibacter	0	0	0	2.69e-03	4.25e-03	3.65e-03
	Deferribacteres;Deferribacteres_Incertae_Sedis;Unknown_Order; Unknown_Family;Caldithrix	3.98e-03	1.08e-02	1.85e-01	1.64e-01	1.74e-01	4.49e-01
	Proteobacteria;Alphaproteobacteria;Rhizobiales;Phyllobacteriaceae ;Nitratireductor	2.79e-02	0	6.86e-03	2.69e-03	8.49e-03	0
	Proteobacteria;Alphaproteobacteria;Rhizobiales;Rhizobiaceae;Shinella	7.96e-03	1.08e-02	0	0	0	0
	Proteobacteria;Betaproteobacteria;Burkholderiales;Comamonadaceae; Alicycliphilus	0	0	0	2.69e-03	0	0
	Proteobacteria;Gammaproteobacteria;Chromatiales;Ectothiorhodospiraceae; Thioalkalivibrio	0	0	0	0	0	3.65e-03
	Proteobacteria;Gammaproteobacteria;Xanthomonadales;Xanthomonadaceae; Stenotrophomonas	3.98e-03	1.08e-02	0	0	0	7.29e-03

Potential ecological function	Bacterial/ Archaeal Taxonomy	Relative abundance (%)					
Nitrification	*Firmicutes;Bacilli;Bacillales;Paenibacillaceae;Brevibacillus*	0	0	0	0	4.25e-05	0
Nitrite oxidation	*Proteobacteria;Betaproteobacteria;Nitrosomonadales;Gallionellaceae; Candidatus_Nitrotoga*	0	0	0	0	4.25e-03	0
	Nitrospirae;Nitrospira;Nitrospirales;Nitrospiraceae;Nitrospira	7.96e-03	0	0	1.34e-02	0	0
Nitrogen fixation	*Proteobacteria;Betaproteobacteria;Burkholderiales;Burkholderiaceae; Burkholderia*	0	1.08e-02	1.37e-02	6.98e-02	5.09e-02	1.24e-01
	Actinobacteria;Actinobacteria;Frankiales;Cryptosporangiaceae;Fodinicola	1.19e-02	0	0	2.69e-03	0	0
	Proteobacteria;Alphaproteobacteria;Rhizobiales;Bradyrhizobiaceae; Bradyrhizobium	1.07e-01	0	0	1.07e-02	4.67e-02	0
	Proteobacteria;Alphaproteobacteria;Rhizobiales;C2U; Pseudoxanthobacter_sp._S50	7.96e-02	1.52e-01	5.49e-02	0	0	0
	Proteobacteria;Alphaproteobacteria;Rhizobiales;Hyphomicrobiaceae; Devosia	0	1.08e-02	6.86e-03	0	0	0
	Proteobacteria;Alphaproteobacteria;Rhizobiales;Xanthobacteraceae; Azorhizobium	3.98e-03	0	0	0	0	0
	Proteobacteria;Alphaproteobacteria;Rhizobiales;Xanthobacteraceae; Pseudoxanthobacter	3.98e-03	5.42e-02	1.37e-02	0	0	0
	Proteobacteria;Alphaproteobacteria;Rhodospirillales;Rhodospirillaceae; Azospirillum	0	0	6.86e-03	0	0	0
	Proteobacteria;Alphaproteobacteria;Rhodospirillales;Rhodospirillaceae; Tistlia	7.96e-03	0	2.06e-02	0	0	0
	Proteobacteria;Gammaproteobacteria;Cellvibrionales;Cellvibrionaceae;	0	0	0	5.37e-03	0	0

**Potential ecological function**	**Bacterial/ Archaeal Taxonomy**	**Relative abundance (%)**					
	Cellvibrio						
	Proteobacteria;Gammaproteobacteria;Enterobacteriales;Enterobacteriaceae; Enterobacter	3.98e-03	0	0	2.42e-02	4.25e-03	3.65e-03
	Proteobacteria;Gammaproteobacteria;Oceanospirillales;Oceanospirillaceae; Marinobacterium	0	0	0	2.69e-03	1.70e-02	2.19e-02
	Firmicutes;Negativicutes;Selenomonadales;Veillonellaceae;Propionispira	3.98e-03	0	0	0	0	0
	Proteobacteria;Betaproteobacteria;Rhodocyclales;Rhodocyclaceae;Azospira	2.79e-02	3.25e-02	7.54e-02	5.37e-03	2.12e-02	4.38e-02
	Firmicutes;Bacilli;Bacillales;Paenibacillaceae;Paenibacillus	5.97e-02	0	0	0	4.25e-03	0
	Proteobacteria;Betaproteobacteria;Rhodocyclales;Rhodocyclaceae;Azoarcus	1.99e-02	0	0	5.64e-02	1.70e-02	0
	Proteobacteria;Betaproteobacteria;Rhodocyclales;Rhodocyclaceae;Azonexus	0	0	0	0	4.25e-03	0
	Proteobacteria;Gammaproteobacteria;Pseudomonadales;Pseudomonadaceae; Pseudomonas	3.98e-03	2.17e-02	2.06e-02	8.33e-02	1.23e-01	7.29e-02
Thiodenitrification	*Proteobacteria;Gammaproteobacteria;Gammaproteobacteria_Incertae_Sedis; Unknown_Family;Thiohalomonas*	0	0	0	5.37e-03	1.27e-02	3.65e-03
Sum of ammonia production and oxidation, nitrite reduction and oxidation,denitrification, nitrification and N fixation		1.24	9.40e-01	1.65	1.42	2.70	1.30

Table S4 continue

**Potential ecological function**	**Bacterial/ Archaeal Taxonomy**	**Relative abundance (%)**					
Fe (II) oxidation	*Nitrospirae;Nitrospira;Nitrospirales;Nitrospiraceae;Leptospirillum*	0	0	2.74e-02	0	8.49e-03	0
	Proteobacteria;Betaproteobacteria;Hydrogenophilales;Hydrogenophilaceae; Thiobacillus	0	0	0	3.76e-02	4.25e-03	0
	Proteobacteria;Zetaproteobacteria;Mariprofundales;Mariprofundaceae; Mariprofundus	1.19e-02	1.08e-02	1.37e-01	4.57e-02	2.12e-02	3.65e-02
	Proteobacteria;Alphaproteobacteria;Rhodobacterales;Rhodobacteraceae; Rhodovulum	0	0	0	0	8.49e-03	0
	Proteobacteria;Alphaproteobacteria;Rhodospirillales;Rhodospirillaceae; Ferrovibrio	3.98e-03	1.08e-02	0	0	0	0
Fe (III) reduction	*Proteobacteria;Betaproteobacteria;Burkholderiales;Comamonadaceae; Albidiferax*	1.59e-02	1.08e-02	0	9.94e-02	7.64e-02	3.65e-03
	Proteobacteria;Gammaproteobacteria;Alteromonadales;Shewanellaceae; Shewanella	7.96e-03	1.08e-02	0	8.06e-02	1.91e-01	4.74e-02
	Firmicutes;Clostridia;Clostridiales;Peptococcaceae;Desulfitobacterium	0	0	0	0	0	1.09e-02
	Proteobacteria;Deltaproteobacteria;Desulfuromonadales;Geobacteraceae; Geoalkalibacter	0	0	0	2.95e-02	1.70e-02	2.19e-02
	Proteobacteria;Deltaproteobacteria;Desulfuromonadales;Desulfuromonadaceae; Desulfuromonas	0	0	0	2.95e-02	8.07e-02	1.82e-02
	Firmicutes;Clostridia;Clostridiales;Clostridiaceae_2;Alkaliphilus	0	0	6.86e-03	0	0	0

Dissimilatory Fe (III) reduction	*Proteobacteria;Alphaproteobacteria;Rhodospirillales;Acetobacteraceae; Acidicaldus*	1.59e-02	1.08e-02	1.37e-02	0	0	3.65e-03
	Proteobacteria;Betaproteobacteria;Burkholderiales;Comamonadaceae; Polaromonas	0	0	0	5.37e-03	0	0
	Proteobacteria;Deltaproteobacteria;Desulfuromonadales;Desulfuromonadaceae; Desulfuromusa	1.19e-02	0	6.86e-03	0	4.25e-03	2.92e-02
	Proteobacteria;Deltaproteobacteria;Desulfuromonadales;Geobacteraceae; Geobacter	0	0	0	1.07e-02	0	3.65e-03
	Deferribacteres;Deferribacteres;Deferribacterales;Deferribacteraceae; Deferribacter	0	0	9.60e-02	0	0	0
	Deferribacteres;Deferribacteres;Deferribacterales;Deferribacteraceae; Geovibrio	0	0	0	0	0	3.65e-03
	Firmicutes;Clostridia;Clostridiales;Peptococcaceae;Thermincola	0	0	0	0	4.25e-03	7.29e-03
	Proteobacteria;Deltaproteobacteria;Bdellovibrionales;Bacteriovoracaceae; Deferrisoma	0	0	0	9.40e-02	6.79e-02	1.82e-01
Sum of Fe (II) oxidation and Fe(III) reduction		7.00e-02	5.00e-02	2.90e-01	4.30e-01	4.80e-01	3.70e-01
Metal tolerant	*Proteobacteria;Alphaproteobacteria;Rhodospirillales;Acetobacteraceae; Acidocella*	0	0	0	2.69e-03	0	0
	Proteobacteria;Betaproteobacteria;Burkholderiales;Burkholderiaceae; Burkholderia	1.19e-02	0	0	2.69e-03	0	0
	Firmicutes;Bacilli;Bacillales;Planococcaceae;Lysinibacillus	1.19e-02	0	0	0	0	7.29e-03
	Actinobacteria;Actinobacteria;Micrococcales;Cellulomonadaceae; Cellulomonas	3.58e-02	0	2.54e-01	5.37e-03	2.55e-02	1.46e-02

	Actinobacteria;Actinobacteria;Corynebacteriales;uncultured; uncultured_Microbacterium_sp.	3.98e-03	2.17e-02	6.86e-03	0	0	7.29e-03
	Actinobacteria;Actinobacteria;Micrococcales;Microbacteriaceae;Leucobacter	1.59e-02	0	0	1.61e-02	8.49e-03	1.09e-02
	Actinobacteria;Actinobacteria;Pseudonocardiales;Pseudonocardiaceae; Amycolatopsis	0	0	0	0	0	1.82e-02
	Proteobacteria;Gammaproteobacteria;Pseudomonadales;Moraxellaceae; Acinetobacter	9.16e-02	1.30e-01	1.03e-01	5.37e-03	2.97e-02	1.09e-02
	Actinobacteria;Actinobacteria;Frankiales;Geodermatophilaceae; Geodermatophilus	3.98e-03	0	0	0	4.25e-03	0
	Actinobacteria;Actinobacteria;Micrococcales;Intrasporangiaceae ;Intrasporangium	0	0	0	0	0	2.92e-02
	Proteobacteria;Betaproteobacteria;Burkholderiales;Comamonadaceae; Caldimonas	0	0	0	5.37e-03	0	0
Metal uptake and accumulation	*Proteobacteria;Betaproteobacteria;Burkholderiales;Comamonadaceae; Variovorax*	7.96e-03	0	0	0	4.25e-03	0
	Actinobacteria;Actinobacteria;Pseudonocardiales;Pseudonocardiaceae; Amycolatopsis	0	0	0	0	0	1.82e-02
	Actinobacteria;Actinobacteria;Micrococcales;Brevibacteriaceae; Brevibacterium	1.19e-02	4.33e-02	0	1.07e-02	1.70e-02	1.17e-01
Sum of metal tolerance, uptake and accumulation reduction		1.90e-01	2.00e-01	3.60e-01	5.00e-02	9.00e-02	2.30e-01
Growth-promoting	*Actinobacteria;Actinobacteria;Corynebacteriales;Tsukamurellaceae; Tsukamurella*	0	0	0	2.69e-03	0	0
	Actinobacteria;Actinobacteria;Corynebacteriales;uncultured; uncultured_Microbacterium_sp.	3.98e-03	2.17e-02	6.86e-03	0	0	7.29e-03

	Actinobacteria;Actinobacteria;Micrococcales;Cellulomonadaceae; Cellulomonas	3.58e-02	0	2.54e-01	5.37e-03	2.55e-02	1.46e-02
	Actinobacteria;Actinobacteria;Micrococcales;Dermabacteraceae; Brachybacterium	3.58e-02	1.08e-02	0	0	0	7.29e-03
	Actinobacteria;Actinobacteria;Micrococcales;Microbacteriaceae; Leifsonia	0	0	0	2.69e-03	0	0
	Actinobacteria;Actinobacteria;Micrococcales;Microbacteriaceae; Microbacterium	5.57e-02	1.63e-01	1.44e-01	2.69e-03	4.25e-03	5.11e-02
	Actinobacteria;Actinobacteria;Pseudonocardiales;Pseudonocardiaceae; Kibdelosporangium	5.57e-02	1.19e-01	2.74e-02	0	0	1.82e-02
	Bacteroidetes;Flavobacteriia;Flavobacteriales;Flavobacteriaceae; Chryseobacterium	3.98e-03	0	0	0	8.49e-03	7.29e-03
	Bacteroidetes;Flavobacteriia;Flavobacteriales;Flavobacteriaceae; Flaviramulus	0	1.08e-02	0	5.37e-03	7.22e-02	3.65e-03
	Bacteroidetes;Sphingobacteriia;Sphingobacteriales;Sphingobacteriaceae; Mucilaginibacter	7.96e-03	0	0	0	0	0
	Proteobacteria;Alphaproteobacteria;Rhizobiales;Rhizobiaceae;Rhizobium	2.79e-02	7.59e-02	1.37e-02	3.49e-02	5.09e-02	2.19e-02
	Proteobacteria;Betaproteobacteria;Burkholderiales;Burkholderiaceae; Burkholderia	1.19e-02	0	0	2.69e-03	0	0
Sum of growth-promoting		2.40e-01	4.00e-01	4.50e-01	6.00e-02	1.60e-01	1.30e-01
Phosphate solubilization	*Actinobacteria;Actinobacteria;Kineosporiales;Kineosporiaceae;Angustibacter*	0	0	0	2.69e-03	0	0
	Actinobacteria;Actinobacteria;Corynebacteriales;uncultured; uncultured_Microbacterium_sp.	3.98e-03	2.17e-02	6.86e-03	0	0	7.29e-03
Polyphosphate accumulation	*Actinobacteria;Actinobacteria;Micrococcales;Intrasporangiaceae; Tetrasphaera*	0	0	9.60e-02	0	8.49e-03	7.29e-03

	Actinobacteria;Actinobacteria;Propionibacteriales;Propionibacteriaceae; Microlunatus	0	0	0	0	0	1.46e-02
	Gemmatimonadetes;Gemmatimonadetes;Gemmatimonadales; Gemmatimonadaceae;Gemmatimonas	0	0	0	2.69e-03	0	3.65e-03
Sum of phosphate solubilization and accumulation		0	2.00e-02	1.00e-01	1.00e-02	1.00e-02	3.00e-02
Amino-acid degradation	*Synergistetes;Synergistia;Synergistales;Synergistaceae;Thermovirga*	0	0	1.73	5.37e-03	4.25e-03	2.19e-02
	Synergistetes;Synergistia;Synergistales;Synergistaceae;Aminobacterium	0	0	0	2.69e-03	4.25e-03	3.65e-03
	Synergistetes;Synergistia;Synergistales;Synergistaceae;Aminomonas	0	0	0	2.69e-03	8.49e-03	3.65e-03
	Synergistetes;Synergistia;Synergistales;Synergistaceae;Lactivibrio	0	0	0	0	0	3.65e-03
Sum of amino-acid degradation		0	0	1.73	1.00e-02	2.00e-02	3.00e-02
Aromatic compounds degradation	*Proteobacteria;Gammaproteobacteria;Thiotrichales;Piscirickettsiaceae; Cycloclasticus*	0	0	0	5.37e-03	0	0
	Proteobacteria;Alphaproteobacteria;Sphingomonadales;Sphingomonadaceae; Sphingomonas	7.96e-03	2.17e-02	1.37e-02	5.37e-03	0	0
	Proteobacteria;Alphaproteobacteria;Sphingomonadales;Sphingomonadaceae; Novosphingobium	1.19e-02	0	0	5.64e-02	1.02e-01	3.28e-02
	Proteobacteria;Gammaproteobacteria;Pseudomonadales;Pseudomonadaceae; Pseudomonas	3.98e-03	2.17e-02	2.06e-02	8.33e-02	1.23e-01	7.29e-02
	Actinobacteria;Actinobacteria;Corynebacteriales;Nocardiaceae;Rhodococcus	7.96e-03	0	1.37e-02	1.61e-02	8.49e-03	1.09e-02
	Proteobacteria;Betaproteobacteria;Rhodocyclales;Rhodocyclaceae;Thauera	1.19e-02	0	0	1.07e-02	4.25e-02	1.82e-02
	Acidobacteria;Holophagae;Holophagales;Holophagaceae;Holophaga	0	0	0	0	8.49e-03	0
	Firmicutes;Clostridia;Clostridiales;Peptococcaceae;Desulfitobacterium	0	0	0	0	0	1.09e-02
	Proteobacteria;Gammaproteobacteria;Vibrionales;Vibrionaceae;Vibrio	3.98e-03	2.17e-02	6.86e-03	2.69e-03	1.70e-02	1.09e-02

	Proteobacteria;Gammaproteobacteria;Oceanospirillales;Oceanospirillaceae; Neptunomonas	0	0	0	5.37e-03	3.82e-02	3.65e-03
	Proteobacteria;Alphaproteobacteria;Rhodobacterales;Rhodobacteraceae; Celeribacter	0	0	0	0	4.25e-03	0
	Proteobacteria;Alphaproteobacteria;Sphingomonadales;Erythrobacteraceae; Erythrobacter	1.19e-02	0	0	1.34e-02	2.97e-02	2.19e-02
	Actinobacteria;Actinobacteria;Corynebacteriales;Mycobacteriaceae; Mycobacterium	1.27e-01	1.52e-01	4.80e-02	2.69e-02	1.70e-02	5.83e-02
	Actinobacteria;Actinobacteria;Corynebacteriales;Nocardiaceae;Gordonia	0	1.08e-02	0	0	0	7.29e-03
Sum of aromatic compounds degradation		1.90e-01	2.30e-01	1.00e-01	2.30e-01	3.90e-01	2.50e-01
Crude-oil degradation	*Actinobacteria;Actinobacteria;Corynebacteriales;uncultured; uncultured_Microbacterium_sp.*	3.98e-03	2.17e-02	6.86e-03	0	0	7.29e-03
	Actinobacteria;Actinobacteria;Micrococcales;Microbacteriaceae; Microbacterium	5.57e-02	1.63e-01	1.44e-01	2.69e-03	4.25e-03	5.11e-02
	Actinobacteria;Actinobacteria;Propionibacteriales;Nocardioidaceae; Nocardioides	1.59e-02	2.17e-02	6.17e-02	8.06e-03	1.70e-02	5.83e-02
	Proteobacteria;Betaproteobacteria;Burkholderiales;Comamonadaceae; Aquabacterium	8.76e-02	1.73e-01	8.91e-02	2.69e-03	0	3.65e-03
	Proteobacteria;Betaproteobacteria;Rhodocyclales;Rhodocyclaceae;Zoogloea	0	0	0	8.06e-03	2.97e-02	3.65e-03
Sum of crude-oil degradation		1.60e-01	3.80e-01	3.00e-01	2.00e-02	5.00e-02	1.20e-01
Environmental pollutant degradation	*Proteobacteria;Alphaproteobacteria;Sphingomonadales;Sphingomonadaceae; Sphingomonas*	7.96e-03	2.17e-02	1.37e-02	5.37e-03	0	0
	Proteobacteria;Alphaproteobacteria;Sphingomonadales;Sphingomonadaceae; Novosphingobium	1.19e-02	0	0	5.64e-02	1.02e-01	3.28e-02

	Firmicutes;Clostridia;Clostridiales;Family_XI;Soehngenia	0	0	0	0	8.49e-03	0
	Proteobacteria;Gammaproteobacteria;Pseudomonadales;Pseudomonadaceae; Pseudomonas	3.98e-03	2.17e-02	2.06e-02	8.33e-02	1.23e-01	7.29e-02
	Actinobacteria;Actinobacteria;Propionibacteriales;Nocardioidaceae; Nocardioides	1.59e-02	2.17e-02	6.17e-02	8.06e-03	1.70e-02	5.83e-02
	Actinobacteria;Actinobacteria;Micrococcales;Intrasporangiaceae; Janibacter	7.48e-01	1.59	5.55e-01	2.69e-03	0	1.09e-02
	Actinobacteria;Actinobacteria;Pseudonocardiales;Pseudonocardiaceae; Pseudonocardia	2.39e-02	0	6.86e-03	0	0	0
	Actinobacteria;Actinobacteria;Pseudonocardiales;Pseudonocardiaceae; Pseudonocardia	2.39e-02	0	6.86e-03	0	0	0
	Actinobacteria;Actinobacteria;Corynebacteriales;Nocardiaceae;Rhodococcus	7.96e-03	0	1.37e-02	1.61e-02	8.49e-03	1.09e-02
	Actinobacteria;Actinobacteria;Micrococcales;Micrococcaceae;Arthrobacter	4.78e-02	1.08e-02	6.86e-02	2.69e-02	4.67e-02	5.11e-02
	Proteobacteria;Gammaproteobacteria;Oceanospirillales;Alcanivoracaceae; Alcanivorax	0	0	0	3.76e-02	7.22e-02	4.01e-02
	Proteobacteria;Deltaproteobacteria;Desulfuromonadales; Desulfuromonadaceae;Pelobacter	0	0	7.54e-02	2.69e-03	4.25e-03	7.29e-03
	Proteobacteria;Betaproteobacteria;Burkholderiales;Burkholderiaceae; Burkholderia	1.19e-02	0	0	2.69e-03	0	0
	Actinobacteria;Actinobacteria;Micrococcales;Microbacteriaceae; Pseudoclavibacter	7.96e-03	0	0	5.37e-03	0	0
	Proteobacteria;Betaproteobacteria;Burkholderiales;Comamonadaceae; Polaromonas	0	0	0	5.37e-03	0	0
	Proteobacteria;Gammaproteobacteria;Cellvibrionales;Cellvibrionaceae; Pseudomaricurvus	0	1.08e-02	0	0	1.70e-02	0

	Proteobacteria;Deltaproteobacteria;Deltaproteobacteria_Incertae_Sedis; Syntrophorhabdaceae;Syntrophorhabdus	1.99e-02	0	0	5.37e-03	4.25e-03	3.65e-03
	Proteobacteria;Alphaproteobacteria;Rhodobacterales;Rhodobacteraceae; Celeribacter	0	0	0	0	4.25e-03	0
	Firmicutes;Clostridia;Clostridiales;Peptococcaceae;Cryptanaerobacter	0	0	0	0	8.49e-03	0
Sum of environmental pollutant degradation		9.30e-01	1.68	8.20e-01	2.60e-01	4.20e-01	2.90e-01

Table S5. Primer sequences for pyrosequencing

#Sample ID	Barcode Sequence	Linker Primer Sequence
R1	TCAGCACG	YMGCCRCGGKAAHACC
R2	TAGACACG	YMGCCRCGGKAAHACC
R3	TGACGCAG	YMGCCRCGGKAAHACC
S1	ATCAGCAG	YMGCCRCGGKAAHACC
S2	TGCTACAG	YMGCCRCGGKAAHACC
S3	AGTGACAG	YMGCCRCGGKAAHACC
